# A closed-loop bioelectronic patch for intelligent blood pressure management

**DOI:** 10.1126/sciadv.adx6438

**Published:** 2025-08-06

**Authors:** Yuyan Zou, Zeguo Chen, Bowen Jin, Linghui Lyu, Yongting Xie, Yuqing Li, Ke Zeng, Siqi Yan, Zhongshi Wu, Wansong Chen, You-Nian Liu, Yanli Zhao

**Affiliations:** ^1^ Hunan Provincial Key Laboratory of Micro and Nano Materials Interface Science, College of Chemistry and Chemical Engineering, Central South University, Changsha, Hunan 410083, China.; ^2^ Department of Cardiovascular Surgery, The Second Xiangya Hospital, Central South University, Changsha, Hunan 410083, China.; ^3^ Institute for Stem Cell and Regenerative Medicine, The Second Affiliated Hospital of Xi'an Jiaotong University, Xi'an, Shaanxi 710004, China.; ^4^ School of Chemistry, Chemical Engineering, and Biotechnology, Nanyang Technological University, 21 Nanyang Link, Singapore 637371, Singapore.

## Abstract

Cardiovascular diseases remain a leading cause of mortality due to passive and delayed drug interventions. This study introduces an intelligent blood pressure management system (BPMS) for real-time monitoring and adaptive intervention through a closed-loop framework integrating sensors, control circuits, and microneedle electrodes. A hierarchical microneedle architecture, featuring gold nanoparticle (Au NP) electrocatalysts and copper-nitrogen–doped carbon nanoribbon (Cu-NC NB) nanozymes, selectively catalyzes the in situ generation of nitric oxide (NO) for vasodilation. To enhance NO delivery, an electroosmotic flow (EOF) mechanism extends the diffusion range up to 4 millimeters through a porous microneedle (PMN) array, enabling effective vascular penetration. In vivo studies in rabbits and pigs confirm that BPMS dynamically regulates NO release in response to fluctuating blood pressure, achieving real-time hemodynamic control. This work pioneers a closed-loop strategy for continuous blood pressure monitoring and on-demand vasodilation, offering a transformative approach to the intelligent management of cardiovascular diseases.

## Teaser

A smart patch monitors blood pressure and releases nitric oxide on demand, offering real-time control for cardiovascular health.

## INTRODUCTION

Cardiovascular-related diseases impose a substantial health care burden with more than 18 million deaths worldwide annually (*1*–*3*). Hypertension as a typical symptom of cardiovascular-related diseases can lead to considerable heart or brain damage and even death in extreme cases (*4*, *5*). Currently, hypertension treatment in clinical settings predominantly relies on oral antihypertensive drugs, such as losartan potassium, bisoprolol, and nifedipine controlled-release and compounded preparations (*6*, *7*). However, this approach requires a strict medication regimen, including drug types, dosages, and durations, which is passive and inconvenient. Nonadherence to this regimen results in ineffective hypertension control or hypotensive events due to overmedication, posing life-threatening risks to patients (*8*). Given the dynamic nature of blood pressure fluctuations, continuous monitoring and prompt intervention are crucial for preventing acute cardiovascular incidents.

Nanobioelectronics integrates nanoscience, biology, and electronics to develop closed-loop medical devices equipped with sensors and controllers (*9*–*12*). These sensors can collect human physiological signals, such as sweat metabolites (*13*, *14*), blood pressure (*15*, *16*), and blood sugar (*17*, *18*), in real-time and establish a stable signal connection with the organism. When the sensor detects abnormal physiological signals, it triggers the actuator to initiate corresponding therapeutic interventions through a closed-loop feedback mechanism (*19*–*22*). Thus, nanobioelectronics can correct abnormal physiological signal through closed-loop devices, which are crucial for achieving intelligent health management. This technology is particularly beneficial for elderly individuals and patients with chronic diseases, dramatically lowering risks associated with traditional untimely methods. However, current research on closed-loop devices is still in its early stages and faces formidable challenges in monitoring and treating cardiovascular diseases such as hypertension. Most existing studies focus on either physiological signal monitoring (*15*, *16*, *23*) or precise drug administration (*24*–*26*), lacking integration through closed-loop feedback, which limits the development of intelligent treatments for cardiovascular diseases.

As an alternative to antihypertensive drugs, nitric oxide (NO) plays a pivotal role in the regulation of normal blood pressure by activating guanylate cyclase, which leads to the relaxation of smooth muscle cells and vasodilation (*27*, *28*). In situ NO generation offers a rapid onset of effect, shortened pharmacokinetic profiles, and high bioavailability with minimal adverse effects (*29*, *30*), thus surpassing the efficacy of passive oral antihypertensive agents. Among the various methods for in situ NO generation, electrochemical synthesis of NO represents a promising approach because of its adjustable release kinetics (*31*, *32*). In particular, the rate of electrocatalytic NO generation can be flexibly modulated by either current or potential, thus offering the possibility for constructing closed-loop bioelectronic devices. This technique can incorporate various electrocatalysts, such as copper(II)-ligand complexes (*33*), iron-based single atoms (*29*), and iron porphyrins (*34*), in conjunction with catheter-encapsulated nitrite reservoirs for biomedical applications. Despite these advancements, electrocatalytic NO generation for biomedical applications confronts daunting challenges. The current electrocatalytic NO generation relies on manual voltage supply, lacking an intelligent response to blood pressure fluctuations. Furthermore, the complex physiological environment may introduce numerous redox-active molecules into the electrocatalytic reactions, leading to side reactions or reactive intermediates with potential adverse effects. In addition, dense tissues can obstruct the penetration and diffusion of gaseous NO, potentially causing its escape from targeted sites after generation. On the basis of these considerations, there is a critical need for electrocatalytic NO generation with selectivity for NO generation and enhanced delivery efficiency for gaseous NO in tissues. To our knowledge, no NO delivery platforms can fulfill these stringent requirements.

**Fig. 1.**
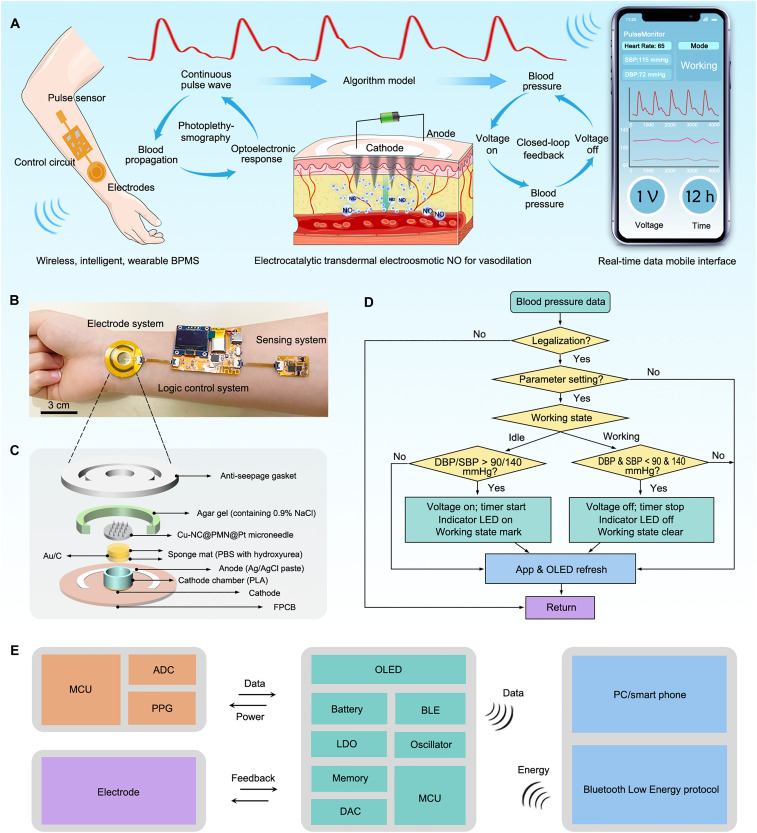
Working principles and system integration of BPMS. (**A**) Schematic diagram of BPMS based on the sensing-feedback principle. (**B**) Optical image of BPMS that includes three subsystems: a pulse sensing system, a logic control system, and an integrated electrode system. (**C**) Scheme of the assembled electrode system consisting of a cathode chamber, PMN, anti-seepage gasket, conductive hydrogel, Ag/AgCl anode, and Au NPs cathode on FPCB. (**D**) Logic flow diagram of the monitoring-feedback manner of the BPMS. (**E**) Block diagram of the main components of the BPMS. DAC, digital-to-analog converter. PC, personal computer. h, hours.

Here, we propose a closed-loop blood pressure management system (BPMS) that integrates continuous blood pressure monitoring with electrocatalytic NO transdermal delivery (Fig. 1A). The system uses photoplethysmography (PPG) to monitor blood pressure, detecting pulse data and generating blood pressure signals via an algorithmic chip. An embedded logic algorithm within the microcontroller unit (MCU) regulates electrode voltage for electrocatalytic NO generation, triggered by blood pressure thresholds (systolic: 140 mmHg; diastolic: 90 mmHg). For in situ electrocatalytic NO generation, gold nanoparticles (Au NPs) serve as two-electron oxygen-reducing electrocatalysts. Selective NO production is facilitated by the hierarchical core-shell structure of the microneedles. Specifically, copper-nitrogen–doped carbon nanoribbons (Cu-NC NBs) are embedded in the microneedles as nanozymes for NO generation, while platinum nanoparticles (Pt NPs) on the outer surface of the microneedles eliminate reactive intermediates and secondary side reactions. To achieve the transdermal NO delivery, we introduced electroosmotic flow (EOF) into the microneedles, which markedly increase the NO transdermal area by four times. Animal models in rabbits and pigs demonstrate that the BPMS continuously monitors blood pressure and intelligently delivers NO in a closed-loop feedback manner for vasodilatation. The wireless configuration of the BPMS facilitates real-time blood pressure management intelligently and automatically. Moreover, this smart point-of-care electronic system is expected to enhance therapeutic outcomes and offer perspectives for cardiovascular health care management.

## RESULTS

### System overview

The wearable patch-type electronic BPMS comprises three subsystems, including a pulse sensing system, a logic control system, and an integrated electrode system (see Fig. 1B). The overall dimensions of the BPMS are 15 cm in length, 40 mm in width, and 5 mm in thickness. All electronic components are meticulously assembled on a 200-μm-thick polyimide, resulting in a flexible printed circuit board (FPCB) that conforms to the skin low modulus. The pulse sensing system operates on the basis of the principle of PPG, allowing it to sense and extract pulse information. These data are then processed by an algorithmic chip, which generates the blood pressure signal (refer to figs. S1 and S2). The logic control system encompasses several key components: (i) An MCU (ESP32-U4WDH) handles data reading, logic processing, and the generation of pulse-width modulation waves. These waves control the voltage between the electrodes. (ii) An antenna facilitates wireless communication via the Bluetooth Low Energy (BLE) protocol. (iii) An organic light-emitting diode (OLED) screen provides visual feedback for blood pressure monitoring. (iv) A light-emitting diode (LED) serves as a visual indicator during electrocatalytic operations. (v) A 100–mA·hour lithium-ion polymer battery, complete with a reserved recharge interface (depicted in fig. S3), powers the system. In the integrated electrode system, a spatially cascaded hybrid cathode is constructed by vertically aligning a Au NP–decorated carbon cloth (Au/C) within the hydroxyurea-loaded sponge layer and the Cu-NC@PMN@Pt porous microneedle (PMN) array atop it, forming a cascade catalytic pathway, where electrocatalysis and enzyme-mimetic catalysis occur sequentially to generate NO. In addition, a Ag/AgCl paste is applied to the FPCB as the counter electrode (Fig. 1C and fig. S4). These three subsystems are interconnected via flexible connectors, forming a wireless, intelligent, wearable BPMS capable of real-time blood pressure monitoring and vasodilation feedback (as illustrated in Fig. 1A).

### Working principles and system integration

PPG is used for continuous, noninvasive blood pressure monitoring due to its high sensitivity to pulsatile blood volume changes and its miniaturized form factor (20 mm by 15 mm) (*35*–*37*). The pulse sensing system adopts a reflection-mode PPG architecture incorporating two pairs of green LEDs (λ ≈ 550 nm) and two high-sensitivity photodetectors (PDs) arranged in a symmetric coplanar layout (see fig. S1). Green light is chosen for its strong hemoglobin absorption and high signal-to-noise ratio at shallow vascular depths (~2 to 3 mm) (*38*). The dual-LED-pair design enables alternating illumination and differential detection, which enhances signal stability, mitigates ambient light interference, and improves illumination uniformity over curved skin surfaces. The two PDs capture backscattered light modulated by pulsatile blood flow from different illumination angles, contributing to spatial averaging and increased robustness. The resulting analog electrical signals are first processed by automatic gain control chip, which adaptively adjusts the amplification level based on signal amplitude. This dynamic gain adjustment compensates for variations in optical intensity due to individual vascular properties, skin pigmentation, motion, and ambient interference, thereby maintaining the signal within an optimal dynamic range and enhancing downstream signal fidelity. These signals then undergo baseline correction, band-pass filtering, and front-end conditioning through a dedicated signal processing chip. The conditioned signals are digitized at 200 Hz by an onboard analog-to-digital converter (ADC), enabling low-power, periodic pulse waveform sampling. Feature extraction is performed by an algorithmic processing chip using pulse wave analysis, where time-domain metrics such as systolic peak time, pulse width, dicrotic notch timing, and augmentation index surrogates are computed. These features are mapped to systolic blood pressure (SBP) and diastolic blood pressure (DBP) values in real time using a multiple linear regression model embedded in firmware. The processed blood pressure data are transmitted to the main logic control unit (MCU; ESP32-U4WDH) via a universal asynchronous receiver/transmitter interface to enable closed-loop regulation (see figs. S1 and S2). To assess the reliability of PPG-based blood pressure estimation, we conducted a validation study using independent subjects under resting conditions. Blood pressure was simultaneously measured using our wearable PPG system and a commercial cuff-based sphygmomanometer (Yuwell S63AR, China). Both SBP and DBP values from the PPG system were highly consistent with cuff-based readings, with an overall Pearson’s correlation coefficient of *r* = 0.93 for SBP and *r* = 0.81 for DBP, confirming excellent linear agreement (fig. S5).

The assembled electrode system is constructed through a hierarchical, multilayered strategy designed to ensure mechanical robustness, spatial confinement of catalysts, and sustained electrocatalytic performance. The system comprises a cathode chamber [polylactic acid (PLA)], a positively charged PMN array (Cu-NC@PMN@Pt), an anti-seepage spacer, a conductive hydrogel, a Ag/AgCl anode, and a nanogold-modified cathode fabricated on FPCB (Fig. 1C and fig. S4). Specifically, the Au NP–deposited carbon cloth is first encapsulated in agar hydrogel, which functions as both an ionically conductive interface and a mechanical binder. This hydrogel-wrapped electrode is then spatially confined within a hydroxyurea-loaded buffer sponge, whose geometry matches the internal dimensions of the cathode chamber, forming a sandwich-like structure that enables two-electron oxygen reduction reactions (ORR) on the Au NP surface (Fig. 1C). A positively charged PMN array (Cu-NC@PMN@Pt) is subsequently mounted atop the chamber, serving dually as a mechanical cap and as the electrochemical interface for transdermal NO delivery. The hierarchical core-shell architecture of Cu-NC@PMN@Pt facilitates selective NO generation following the ORR cascade. In this structure, Cu-NC NBs are embedded in the microneedles as nanozymes for NO generation, while Pt NPs coated on the microneedle surfaces function as reactive intermediate scavengers, mitigating side reactions. The mesoporous structure of the microneedles provides abundant transport channels for reactants and NO release. An anti-seepage gasket with medical adhesive ensures effective electrical isolation between the electrodes and secures the patch to the skin. A soft conductive hydrogel is employed at the anode-skin interface to minimize contact impedance and enhance electrochemical coupling between the Ag/AgCl electrode and the epidermis. The electrode ports are connected to the output terminals of a logic control system, which regulates the voltage-driven EOF across the PMNs to accelerate NO transdermal transport. The microcontroller is programmed with a feedback algorithm that initiates NO release when blood pressure exceeds predefined thresholds (SBP ≥ 140 mmHg; DBP ≥ 90 mmHg) (Fig. 1D). Upon triggering, a set voltage is applied across the electrode pair, activating the cascade reaction for controlled NO generation over a fixed duration. Once NO-induced vasodilation reduces blood pressure to normal levels, the applied voltage is automatically terminated (fig. S6). This design enables the BPMS to function as a real-time closed-loop system with intelligent responsiveness to blood pressure fluctuations. All physiological data are wirelessly transmitted to a smartphone in real time via Bluetooth for continuous monitoring. In addition, the BPMS supports user-defined reverse mode operation, allowing manual control of NO release through a mobile interface (Fig. 1E and fig. S6).

### Validation of electrocatalytic ORR to H_2_O_2_ generation and NO cascade production of Au NPs

**Fig. 2.**
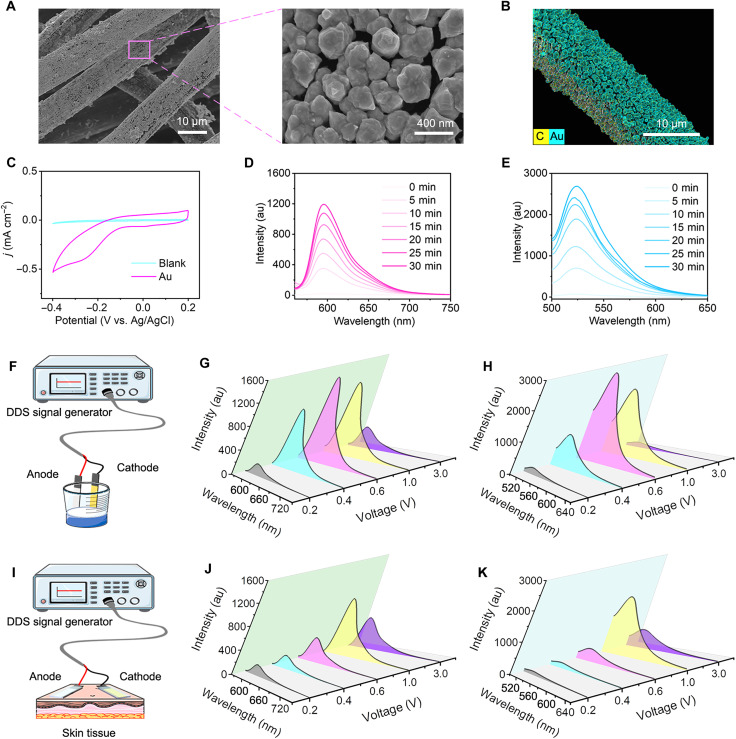
Validation of electrocatalytic ORR to H_2_O_2_ generation and NO cascade production of Au NPs. (**A**) Scanning electron microscopy (SEM) image of electrochemical deposition Au NPs on carbon cloth. (**B**) Elemental mapping of Au NPs on carbon cloth. (**C**) CVs obtained on bare carbon cloth and Au NPs. Fluorescence spectra of (**D**) H_2_O_2_ and (**E**) NO generated from Au NPs under −0.3 V versus Ag/AgCl at different electrolysis times. (**F**) Schematic diagram of a two-electrode DC power supply solution system. Fluorescence spectra of (**G**) H_2_O_2_ and (**H**) NO electrolyzed by Au NPs for 30 min at different voltages in two-electrode DC power solution system. (**I**) Schematic diagram of a two-electrode DC power biological tissue interface system. Fluorescence spectra of (**J**) H_2_O_2_ and (**K**) NO electrolyzed by Au NPs for 30 min at different voltages in two-electrode DC power biological tissue interface system. a.u., arbitrary unit. DDS, direct digital synthesis.

In the electrocatalytic NO generation, Au NPs were selected as the catalyst for the two-electron ORR because of their superior ORR activity and exceptional biocompatibility (*39*, *40*). Au NPs with a diameter of 300 nm were uniformly anchored onto the surface of the carbon fibers via an electrochemical deposition method (Fig. 2, A and B). The electrochemical behavior of the Au NPs was studied using cyclic voltammetry (CV) in phosphate-buffered saline (PBS; 10 mM, pH 7.4). As shown in Fig. 2C, the capacitive current density achieved by the Au NPs reached −0.44 mA cm^−2^ at −0.3 V (versus Ag/AgCl), which is a 22-fold increase compared to the bare carbon cloth (−0.02 mA cm^−2^). The CV curve of Au NPs exhibited a distinct redox peak, indicative of the reduction of O_2_. The electrocatalytic O_2_ reduction to H_2_O_2_ was monitored using Amplex Red as a probe (*41*). During constant potential electrolysis at −0.3 V (versus Ag/AgCl), the H_2_O_2_ generation by the Au NPs gradually increased over time, reaching a yield of 5 μM within 30 min. In contrast, the H_2_O_2_ yield from the bare carbon cloth electrolyte was negligible (<0.3 μM) (Fig. 2D and fig. S7). Hydroxyurea is a well-documented precursor for H_2_O_2_-activated NO generation in the presence of horseradish peroxidase (HRP) (*42*). After verifying the electrocatalytic H_2_O_2_ generation, we quantified the cascade production of NO using 4-amino-5-methylamino-2′,7′-difluorofluorescein (DAF-FM) as a fluorescent probe (*27*, *28*). After 30 min of electrolysis, the NO yield from the Au NPs was up to 2.5 μM, while NO generation from bare carbon cloth was barely detected (Fig. 2E and fig. S8). Therefore, Au NPs can act as ORR electrocatalysts at the cathode for effective NO generation.

To enhance the portability of electrocatalytic NO generation, we explored an alternative to the conventional electrochemical workstation using direct current (DC) power (as depicted in Fig. 2F). In this approach, Au NPs were deposited onto carbon cloth as the cathode, and Ag/AgCl was printed onto carbon cloth as the anode. When the DC voltage is set at 0.6 V, the cathode exhibits the highest electrocatalytic efficiency for ORR-driven NO generation (Fig. 2, G and H). To investigate the electrocatalytic activity within skin tissue, we embedded both the cathode and anode in an agarose hydrogel (2% w/v) and attached it to pork skin tissue (Fig. 2I). NO generation remained feasible even in this skin tissue context. However, because of the resistance posed by the skin tissue, the optimal voltage for ORR-driven NO generation increased to 1 V (Fig. 2, J and K).

### Construction and characterization of Cu-NC@PMN@Pt

**Fig. 3.**
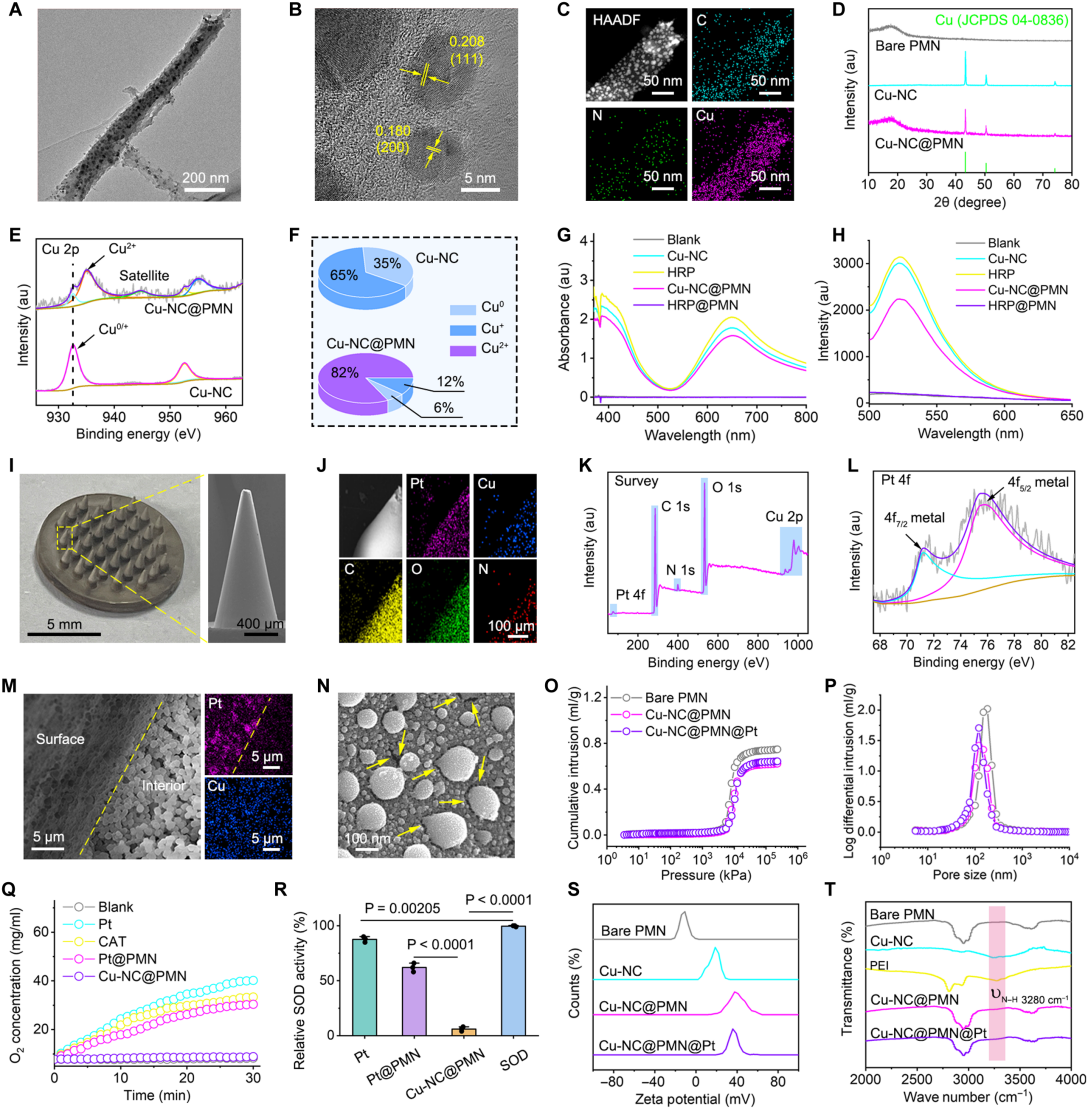
Construction and characterization of Cu-NC@PMN@Pt. (**A**) TEM image of Cu-NC NBs. (**B**) HR-TEM image of Cu-NC NBs. (**C**) Elemental mapping of Cu-NC NBs. (**D**) X-ray diffraction patterns. (**E**) Cu 2p HR-XPS spectra of Cu-NC NBs and Cu-NC@PMN. (**F**) Statistical results of valence fractions of Cu in Cu-NC NBs and Cu-NC@PMN. (**G**) Ultraviolet-visible (UV-vis) absorption spectra of oxidized TMB under different treatments. (**H**) Fluorescence spectra of cascade catalyzed production of NO under different treatments. (**I**) Representative photograph and enlarged cross-sectional SEM image of Cu-NC@PMN@Pt array. (**J**) Elemental mapping of Cu-NC@PMN@Pt. (**K**) XPS spectra of Cu-NC@PMN@Pt. (**L**) HR-XPS spectra of Pt 4f. (**M**) SEM image of the interior-surface junction and the corresponding mapping of Pt and Cu elements of Cu-NC@PMN@Pt. (**N**) SEM image of the Cu-NC@PMN@Pt surface; yellow arrows indicate the location of the pores. (**O**) Piezomercury curves of cumulative intrusion versus pressure and (**P**) pore size distribution curves for PMNs. (**Q**) O_2_ generation–time curves under different treatments. Both Pt NPs and natural catalase (CAT) were used at 10 μg ml^−1^. (**R**) The bar charts of SOD-like activity of Pt, Pt@PMN, and Cu-NC@PMN; natural SOD (100 U ml^−1^) was used as a positive control and defined as 100% activity. (**S**) Zeta potentials of bare PMN, Cu-NC NBs, Cu-NC@PMN, and Cu-NC@PMN@Pt. (**T**) Fourier transform infrared (FTIR) spectra of bare PMN, Cu-NC NBs, polyethyleneimine (PEI), Cu-NC@PMN, and Cu-NC@PMN@Pt. Data in (R) are expressed as means ± SD. *n* = 3 independent samples. One-way analysis of variance (ANOVA) and Tukey’s post hoc test were used, and *P* values are labeled on the graphs. *P < 0.05; **P < 0.01; ***P < 0.001; ****P < 0.0001. HAADF, High-Angle Angular Dark-Field.

To realize transdermal NO generation with dual requirements of electrocatalytic cascade and reusability, we engineered the microneedles as solid, porous, nonswelling, and insoluble structures using biocompatible poly(glycidyl methacrylate) (PGMA). The porous architecture was tailored via monomer-porogen ratio optimization to balance catalytic surface area and mechanical robustness (*17*, *43*, *44*). Six microneedle geometries were fabricated and evaluated, varying in tip shape (conical versus quadrangular pyramidal) and length (1, 2, or 3 mm). Ex vivo porcine skin insertion confirmed that 2-mm conical microneedles provided the optimal balance between penetration depth and structural stability, while avoiding bleeding or excessive discomfort (fig. S9). This geometry was thus adopted as the standard for the Cu-NC@PMN@Pt microneedle platform. Given that microneedles integrated with natural HRP experience complete enzymatic inactivation, we sought to address this challenge using an artificial enzyme with superior stability and durability. The Cu-NC NBs with peroxidase (POD)–mimic activity were synthesized using a one-pot hydrothermal method. Transmission electron microscopy (TEM) reveals a two-dimensional (2D) sheet-like nanostructure of Cu-NC NBs anchored with metallic Cu (Fig. 3A). High-resolution transmission electron microscopy (HR-TEM) further confirmed characteristic lattice spacings of 0.208 and 0.180 nm, corresponding to the (111) and (200) planes of Cu nanocrystals, respectively (Fig. 3B). Elemental mapping results demonstrate uniform distribution of C, N, and Cu in Cu-NC NBs (Fig. 3C). The crystal structure of Cu-NC NBs aligns with the Cu [Joint Committee on Powder Diffraction Standards (JCPDS) 04-0836] and remains intact after the integration with microneedles (Fig. 3D). High-resolution x-ray photoelectron spectroscopy (HR-XPS) analysis was performed to investigate the valence of Cu in Cu-NC NBs. The atomic ratio of Cu^0^ and Cu^+^ in the Cu-NC NBs was about 35 and 65%, respectively. After integration within microneedles, the proportion of Cu^0^ and Cu^+^ species individually decreases to 6 and 12%. Notably, 82% of Cu undergoes oxidation into Cu^2+^ species during free radical polymerization of the microneedles (Fig. 3, E and F, and fig. S10).

The POD-mimic activity of Cu-NC@PMN was evaluated with 3,3′,5,5′-tetramethylbenzidine (TMB) as a colorimetric probe (*45*). Cu-NC NBs can act as POD-like nanozymes to catalyze the TMB oxidation into dimer charge-transfer complex with blue color. According to the kinetic study (fig. S11), the maximum rate of reaction (*V*_max_) and Michaelis constant (*K*_m_) were measured to be 0.093 ± 0.008 μM s^−1^ and 1.43 ± 0.21 mM, respectively, which were comparable to those of natural HRP (*46*). During microneedle fabrication, radical oxidation renders up to ~100% of HRP inactive. In contrast, Cu-NC NBs retain 87% of their POD-like activity within the microneedles, making them a robust alternative to HRP (Fig. 3G and fig. S12). Furthermore, we investigated the ability of Cu-NC@PMN in catalyzing NO generation. About 2 μM NO was generated by Cu-NC@PMN within 30 min under DC voltage of 1 V (Fig. 3H). Therefore, Cu-NC NBs with robust POD-mimic activity can form a cascade with the electrocatalysis of Au NPs for NO generation.

Reactive intermediate •O_2_^−^ and H_2_O_2_ are usually generated in ORR (*47*). Pt NPs are documented with multiple antioxidant mimetic enzyme activity, such as catalase (CAT) and superoxide dismutase (SOD) (*48*, *49*). To ensure electrocatalysis biosafety, we grew Pt NPs in situ on the Cu-NC@PMN surface, resulting in Cu-NC@PMN@Pt. Pt NPs were expected to prevent excess H_2_O_2_ and •O_2_^−^ from spilling out of the side surfaces of the PMN, thereby minimizing oxidative damage to surrounding normal tissues. The representative Cu-NC@PMN@Pt structure consists of an array of 37 conical PMNs (with a height of 2000 μm, base diameter of 800 μm, and tip diameter of 30 μm) on a porous substrate (ϕ10 mm by 1 mm in thickness) (Fig. 3I). Elemental mapping images reveal a homogeneous distribution of Pt, C, N, O, and Cu elements, consistent with the XPS survey spectra (Fig. 3, J and K). XPS analysis of Pt 4f confirms the presence of zero-valent Pt coating on the microneedles (Fig. 3L). The crystallinity of Pt matches the standard card for elemental Pt (JCPDS 04-0802) (fig. S13). The junction of the interior surface of Cu-NC@PMN@Pt was detected through scanning electron microscopy (SEM). Notably, Pt NPs only grow on the surface of the microneedles and separate from the inner Cu-NC NBs (Fig. 3M). Such core-shell structure spatially circumvents the interference between the enzyme activity of Pt NPs and Cu-NC NBs. In addition, numerous pores were randomly distributed on the surface and interior of the Cu-NC@PMN@Pt (Fig. 3, M and N). The pore size distribution of the microneedles was measured using mercury intrusion method. The bare PMN exhibited an average pore size of 112 nm, which slightly decreased to 94 nm upon the incorporation of Cu-NC NBs and Pt NPs (Fig. 3, O and P).

The CAT-mimic activity of Pt NPs was determined by dissolved oxygen meter. According to the elevation of dissolved oxygen concentration, the catalytic kinetics of Pt NPs was found to align well with the Michaelis-Menten model. In particular, *V*_max_ and *K*_m_ were determined to be 0.085 ± 0.008 mg liter^−1^ s^−1^ and 0.029 ± 0.008 M, respectively (fig. S14). Furthermore, we investigated the CAT-mimic activity of Pt nanocoating using H_2_O_2_ as the substrate. A large number of O_2_ bubbles was continuously generated in the H_2_O_2_ solution, indicative of the CAT-mimic activity of Pt nanocoating on the microneedle surface (Fig. 3Q and fig. S15). In terms of scavenging •O_2_^−^, the SOD-mimic activity of Pt NPs was calculated to be 522 U mg^−1^ (fig. S16). The Pt nanocoating on the microneedles retained up to 72% of the SOD-mimic activity observed in Pt NPs (Fig. 3R and fig. S17). In contrast, neither CAT-mimic activity nor SOD-mimic activity was detected in Cu-NC@PMN (Fig. 3, Q and R, and figs. S15 and S17). Together, the Cu-NC@PMN@Pt was successfully constructed for NO generation with the cascade of electrocatalysis of Au NPs and POD-like activity of Cu-NC NBs. Meanwhile, the outer coating of Pt NPs with CAT/SOD-like activity effectively prevents excessive reactive oxygen species (ROS) leakage.

### Evaluation of EOF

Gaseous NO molecules tend to escape from the tissue postgeneration. In addition, intrinsic tissue pressure and blood pressure hinder the diffusion of dissolved NO from microneedles into tissue (*50*). To circumvent these barriers in transdermal NO delivery, we used EOF for the mass transfer during electrocatalytic NO generation. EOF is the movement of a liquid through a microchannel driven by an applied potential (*51*). Under the action of the electric field, the net moving charge near the solid interface in the solution is transferred by the coulomb force, propelling the liquid to move in a directional manner (*43*, *52*). Thus, EOF is anticipated to enhance the transdermal delivery of dissolved NO during electro-enzymatic cascade generation. The direction of EOF hinges on two critical factors: the orientation of the electric field and the type of charge immobilized within the microchannel. To facilitate NO flow from the cathode to the anode, the microneedles were required to be functionalized with positive charges (fig. S18). These positively charged inner pore surfaces electrostatically adsorb anionic counterions such as Cl^−^ and HPO_4_^2−^, forming a stable electric double layer (EDL). When an external electric field is applied, the anions within the EDL migrate electrophoretically toward the anode, and, through viscous coupling, they drag surrounding solvent molecules, generating a bulk EOF that propels dissolved NO toward the skin interface for transdermal delivery (fig. S18). The surface charge of the microneedle channels was determined using phase-analysis light scattering technology. As shown in Fig. 3S, bare PMN inherently carries a negative charge because of the enrichment of epoxy groups and ester groups. However, upon decorating the microneedles with positively charged Cu-NC NBs and polyethyleneimine (PEI), the surface charge of the resulting Cu-NC@PMN@Pt microneedles was reversed to +39.8 ± 1.6 mV. Fourier transform infrared (FTIR) spectroscopy analysis confirms that the PEI coating introduces abundant amine moieties into the microneedle channels, resulting in a strongly positive charge (Fig. 3T).

**Fig. 4.**
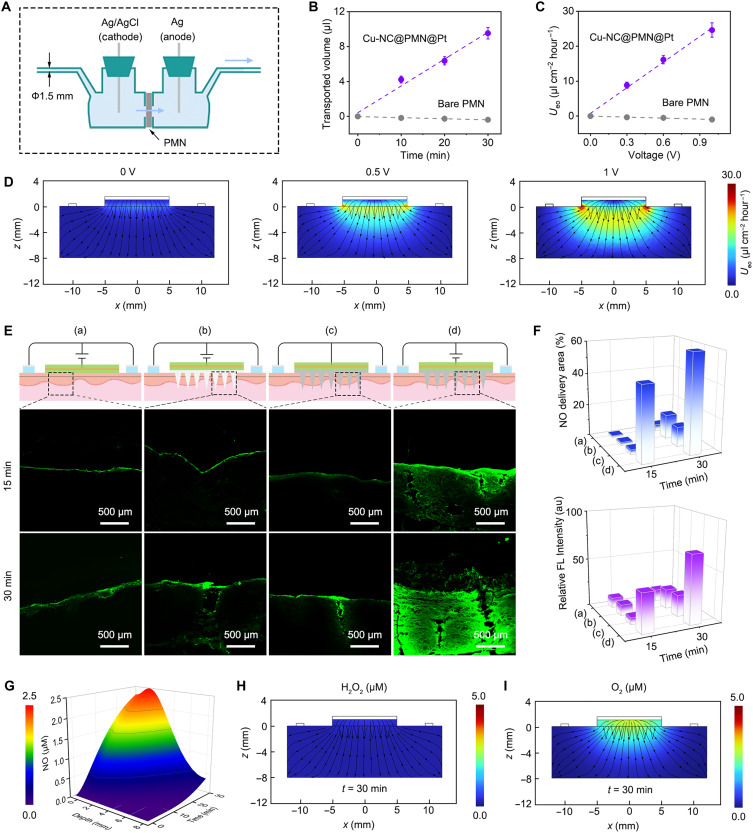
Evaluation of EOF. (**A**) Schematic diagram of an H-type closed electrolytic cell with horizontal capillary tubes for measuring the water migration flux through PMN at a DC supply. (**B**) Volume of water migration as a function of time under 1-V_DC_ voltage. (**C**) Flow velocity of EOF as a function of voltage applied for a bare PMN and Cu-NC@PMN@Pt. (**D**) The EOF velocity of Cu-NC@PMN@Pt at different voltages by finite element simulation. (**E**) Cross-sectional fluorescence images of pork skin through electro-enzymatic catalysis NO transdermal application under various treatments: (a) *E*, (b) bare PMN + *E*, (c) Cu-NC@PMN@Pt, and (d) Cu-NC@PMN@Pt + *E*. (**F**) Quantitative statistics of fluorescence area and relative fluorescence (FL) intensity of NO in porcine skin sections by ImageJ. (**G**) Simulated spatial and temporal evolution of NO. Finite element cross-section simulated concentration of (**H**) H_2_O_2_ and (**I**) O_2_ generation after 30 min of electrolysis by Au-hybridized Cu-NC@PMN@Pt cathode.

To intuitively assess the strength of EOF, we used an H-type closed electrolytic cell equipped with horizontal capillary tubes. This setup allows us to measure the water migration flux through the PMN under a DC supply (Fig. 4A). The flux migrating distance from the cathode side was almost equal to the increment of water on the anode side, demonstrating water migration from the cathode to the anode via the positively charged PMN (fig. S19). The water flux of Cu-NC@PMN@Pt microneedles driven by EOF is proportional to time and voltage. Specifically, the water flux can reach ~10 μl at a DC voltage of 1 V within 30 min. In contrast, the water flux of the naked PMN under the same conditions is negligible because of the lack of EOF (Fig. 4, B and C). The electroosmotic transdermal delivery flux rate of Cu-NC@PMN@Pt microneedles was simulated with COMSOL Multiphysics. According to the simulation results, the EOF rate was barely detected (<0.01 μl cm^−2^ hour^−1^) in the absence of applied voltage. However, the flow rate increased to 25 μl cm^−2^ hour^−1^ at 1 V in a voltage-dependent manner, which is in accordance with the experimental results (Fig. 4, C and D).

To visually observe the EOF within the skin, we investigated the transdermal delivery of NO through electro-enzymatic catalysis in porcine skin using a preloaded DAF-FM probe. NO is blocked on the outer surface of the skin because of its poor permeability to the stratum corneum. When bare PMN was inserted into the skin, NO penetration remained confined to the microneedle region, even after exposure to a DC voltage of 1 V for 30 min. By contrast, for the positively charged microneedles with EOF, the skin penetration of NO covered the entire skin, which was four times the area of bare PMN penetration. Notably, in the absence of applied voltage, the penetration area of NO from the positively charged microneedle to the skin remains limited to the microneedle region (Fig. 4, E and F). Therefore, the EOF in positively charged microneedles effectively facilitates the transdermal delivery of NO through the microneedle channels under a DC voltage in the 2D longitudinal and transverse axes. In addition, a 3D finite element analysis was performed to simulate the spatial distribution of various substance concentrations under a 1-V DC voltage. After the insertion of Cu-NC@PMN@Pt microneedles into the skin and subsequent electrolytic treatment for 30 min, the local NO concentration reached ~1.4 μM at a tissue depth of 4 mm (Fig. 4G). Concurrently, the excess H_2_O_2_ was decomposed by Cu-NC@PMN@Pt microneedles into O_2_ (Fig. 4, H and I). Considering that capillary vessels typically reside at depths of 1 to 2 mm (*53*), Cu-NC@PMN@Pt microneedles facilitated by EOF can adequately deliver NO into the blood vessels, circumventing the residual presence of ROS (Fig. 4, E to I).

### Evaluation of biocompatibility

**Fig. 5.**
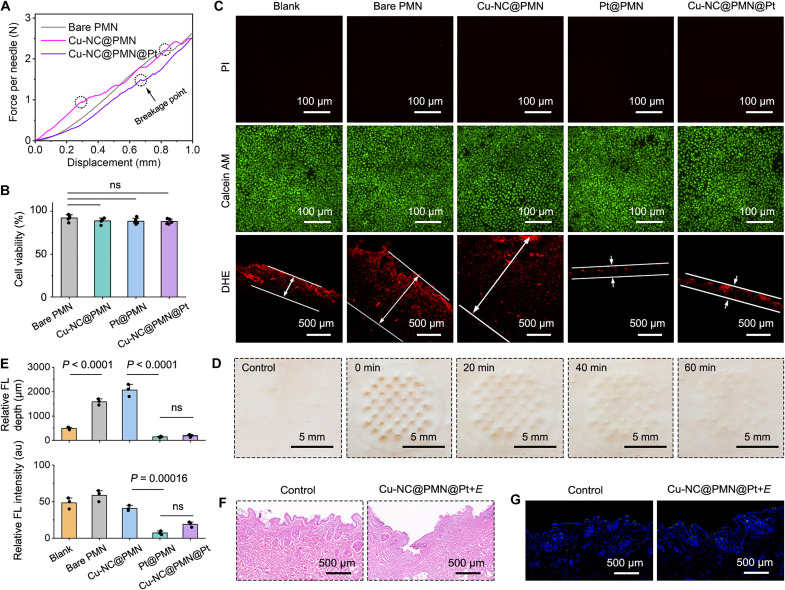
Evaluation biocompatibility of Cu-NC@PMN@Pt. (**A**) Mechanical properties of PMNs under compressive loading. (**B**) Cell viability of HUVECs after incubation with medium impregnated with PMNs (0.1 g ml^−1^) for 24 hours. (**C**) Fluorescence images of live/dead HUVEC cells cultured for 24 hours in medium impregnated with PMNs (0.1 g ml^−1^) and tissue ROS levels after various treatments under 1 V_DC_ ORR for 30 min. (**D**) Rabbit skin recovery process after 12 hours of Cu-NC@PMN@Pt treatment. (**E**) Corresponding fluorescence depths and relative fluorescence intensity of dihydroethidium (DHE). (**F**) Hematoxylin and eosin (H&E) staining and (**G**) TUNEL staining of untreated skin or Au-hybridized Cu-NC@PMN@Pt–treated skin under 1 V_DC_ for 2 hours. Data in (B) are expressed as means ± SD; *n* = 5 independent samples. Data in (E) are expressed as means ± SD; *n* = 3 independent samples. One-way ANOVA and Tukey’s post hoc test were used, and *P* values are labeled on the graphs. *P < 0.05; **P < 0.01; ***P < 0.001; ****P < 0.0001. ns denotes no significant difference (*P* > 0.05).

To ascertain the mechanical robustness of PMNs for skin penetration, we conducted a microcompression test. It is established that microneedles must endure a minimum compression force of 0.4 N to penetrate the skin without yielding mechanically (*54*). As illustrated in Fig. 5A, the breakage point of Cu-NC@PMN@Pt microneedles was 1.5 N, which was 3.75-fold greater than the requisite 0.4 N. Thus, Cu-NC@PMN@Pt microneedles possess adequate mechanical strength for skin puncture. The biocompatibility of Cu-NC@PMN@Pt microneedles as a skin patch is another important factor to evaluate before their biomedical applications. Cytotoxicity evaluation on human umbilical vein endothelial cells (HUVECs) reveal that cell viability exceeds 85% after 24 hours of exposure to PMN leachate (Fig. 5B). In addition, all of the HUVECs were living and stained by calcein acetoxymethyl ester (AM) with green fluorescence (Fig. 5C), indicative of the negligible cytotoxicity of Cu-NC@PMN@Pt microneedles. To evaluate the skin recovery ability, we treated rabbits with Cu-NC@PMN@Pt for 12 hours. After the removal of Cu-NC@PMN@Pt, the array of skin micropores penetrated by Cu-NC@PMN@Pt healed completely within 60 min without erythema or lesions (Fig. 5D).

Subsequently, we verified the ROS scavenging ability of Pt nanocoating in skin tissues with dihydroethidium (DHE) as a ROS probe (*55*). Skin treated with Cu-NC@PMN microneedles displays pronounced red fluorescence from DHE, indicating ROS leakage (Fig. 5, C and E). In contrast, the DHE fluorescence was insignificant in the skin after the treatment of Pt@PMN and Cu-NC@PMN@Pt. Therefore, Pt nanocoating on PMN could act as antioxidant enzyme to scavenge leaked ROS. Furthermore, the local skin tissues were collected and histologically examined by hematoxylin and eosin (H&E) staining. The skin tissue preserves structural integrity in comparation to the untreated skin, and few inflammatory cells were found after the electro-enzymatic catalysis of Cu-NC@PMN@Pt microneedles (Fig. 5F). In addition, the terminal deoxynucleotidyl transferase–mediated deoxyuridine triphosphate nick end labeling (TUNEL) staining of skin slices reveals negligible apoptosis of cells after the treatment of Cu-NC@PMN@Pt microneedles under 1-V DC voltage (Fig. 5G). Collectively, these findings underscore the high mechanical strength and superior biocompatibility of Cu-NC@PMN@Pt microneedles for transdermal NO delivery.

### Validation of vasodilation

**Fig. 6.**
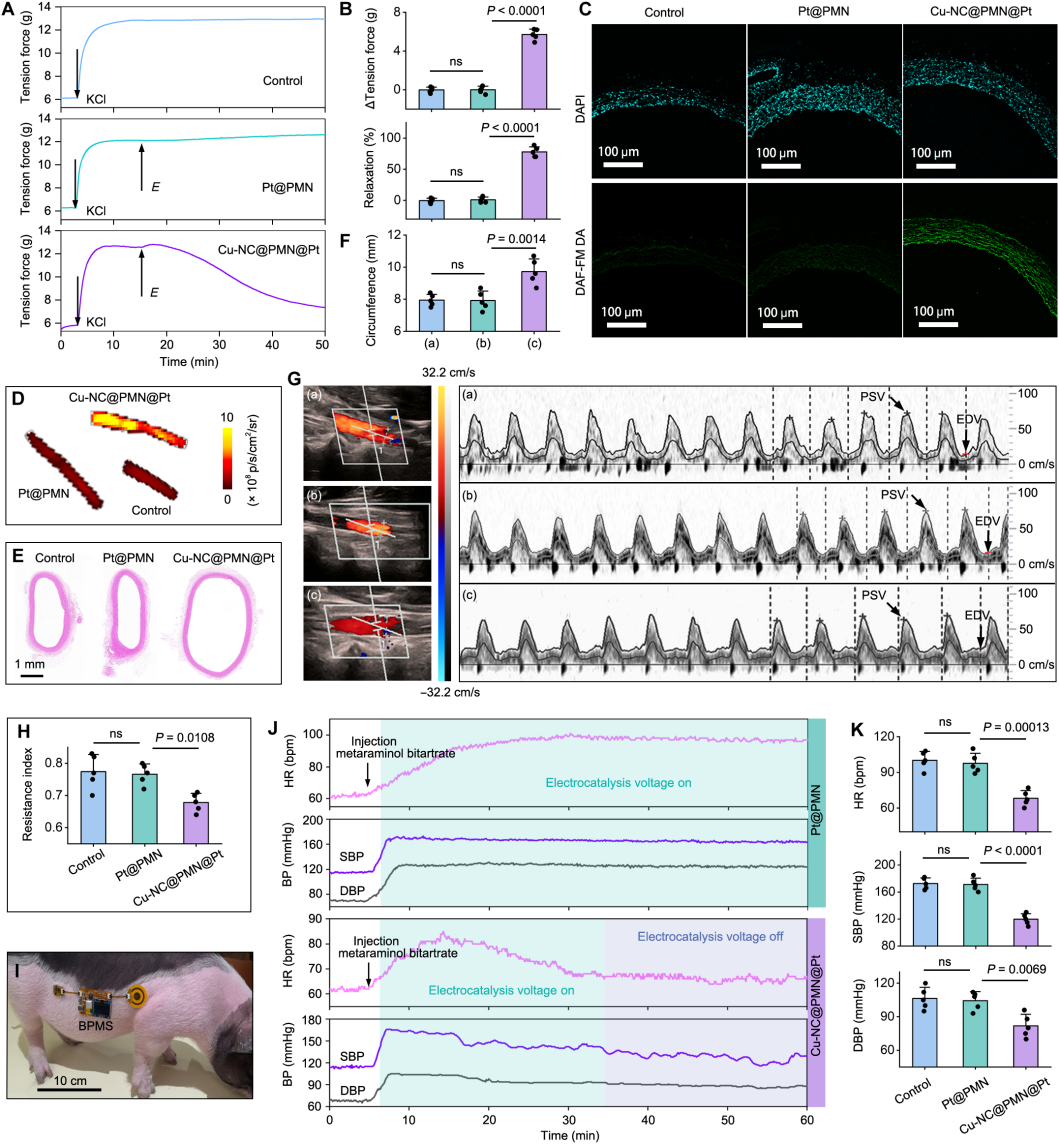
Validation of vasodilation. (**A**) Representative tension curves of ex vivo vasodilatation over time in the case of BPMS assembled with Pt@PMN or Cu-NC@PMN@Pt at 1 V_DC_. (**B**) Bar charts of tension reduction (ΔTension force) and vasodilation (% relaxation) obtained from the experiments shown in (A). Group: (a) control, (b) Pt@PMN, and (c) Cu-NC@PMN@Pt. (**C**) Fluorescence confocal images of frozen sections with DAF-FM DA and 4′,6-diamidino-2-phenylindole (DAPI) staining of vascular tissue; rabbits were administrated BPMS assembled with Pt@PMN or Cu-NC@PMN@Pt for 30 min at 1 V_DC_. (**D**) Fluorescence images of bulk blood vessels stained with DAF-FM DA. (**E**) Representative H&E images and (**F**) corresponding histograms of vessel circumference; rabbits were administrated BPMS assembled with Pt@PMN or Cu-NC@PMN@Pt for 30 min at 1 V_DC_. (**G**) Spectral Doppler imaging of ventral artery after different treatments: (a) control, (b) Pt@PMN-coupled BPMS, and (c) Cu-NC@PMN@Pt–coupled BPMS. EDV, end-diastolic velocity; PSV, peak systolic velocity. (**H**) Statistical results of RI from (G). (**I**) Photograph of a pig administered with BPMS. (**J**) Heart rate (HR) and blood pressure measured during a 1-hour continuous monitoring-feedback intervention by Pt@PMN- or Cu-NC@PMN@Pt–coupled BPMS. (**K**) Statistical results of HR and blood pressure after administration with Pt@PMN- or Cu-NC@PMN@Pt–coupled BPMS. Data in [(B), (F), (H), and (K)] are expressed as means ± SD. *n* = 5 biologically independent samples. One-way ANOVA and Tukey’s post hoc test were used, and *P* values are labeled on the graphs. *P < 0.05; **P < 0.01; ***P < 0.001; ****P < 0.0001. ns denotes no significant difference (*P* > 0.05).

The capacity of BPMS in the electro-enzymatic catalysis of NO generation for vasodilation was investigated ex vivo. A fresh 5-mm-wide thoracic aortic ring was connected to a force transducer in the Krebs-Henseleit buffer. The PMN was inserted into rabbit skin, followed by preconstriction of the arterial loop to simulate hypertension. Upon reaching tension equilibrium, electrocatalysis was initiated at 1 V, and the change in tension over time was recorded (Fig. 6, A and B, and figs. S20 and S21). When Cu-NC@PMN@Pt was used as transdermal microneedles, the tension of the arterial ring rapidly decreased by 6 g, resulting in a relaxation rate of 75%. In contrast, minimal change was observed in the control group or when Pt@PMN was used in the experiments. Thoracic arteries from rabbits were harvested after electrocatalysis for 30 min. These arteries were stained with DAF-FM diacetate (DA) to validate the ex vivo production of NO. Compared to the control group, Cu-NC@PMN@Pt–coupled BPMS results in an eightfold elevation of the DAF-FM DA fluorescence in the vessel slices, whereas Pt@PMN-coupled BPMS shows limited fluorescence signal (Fig. 6C and fig. S22). The overall fluorescence intensity of the bulk vessel significantly rose after treatment with Cu-NC@PMN@Pt–coupled BPMS, particularly at the penetrating sites (Fig. 6D and fig. S22). Furthermore, the circumference of the blood vessel within the section tissue was measured to access the degree of ex vivo vasodilation. According to H&E staining results of the arterial rings (Fig. 6, E and F), the vessel circumference increased significantly from 7.94 ± 0.32 to 9.72 ± 0.71 mm after treatment with Cu-NC@PMN@Pt–coupled BPMS.

Encouraged by the ex vivo findings, we investigated the in vivo performance of Cu-NC@PMN@Pt–coupled BPMS for vasodilation in a monitoring-feedback manner. The vascular resistance index (RI) is an important hemodynamic index for evaluating blood perfusion, which directly reflects the magnitude of vascular resistance and indirectly assesses the degree of vasodilation (*56*). In view of this, spectral Doppler ultrasonography was applied to evaluate blood flow rates and calculate RIs. In rabbits, the time-averaged blood flow velocity of the ventral artery decreased from 22.29 ± 6.75 to 12.04 ± 5.38 cm s^−1^ after intervention with Cu-NC@PMN@Pt–coupled BPMS compared with the control group (Fig. 6G). RI is measured by Doppler sonography in a ventral artery as the difference between the peak systolic and end-diastolic blood velocities divided by the peak systolic velocity. The RI was significantly reduced from 0.77 ± 0.05 to 0.68 ± 0.03 after Cu-NC@PMN@Pt–coupled BPMS administration (Fig. 6, G and H). In contrast, rabbits treated with Pt@PMN-coupled BPMS still retained high RI levels (0.76 ± 0.03). This result indicates the ability of BPMS to reduce vascular resistance through vasodilation.

To construct the acute hypertension model, we administered an intramuscular injection of metaraminol bitartrate at a dosage of 0.1 mg kg^−1^ to Bama pigs, inducing blood pressure elevation (*57*). The successful construction of the acute hypertensive pig model was indicated by maintaining SBP ≥ 140 mmHg and DBP ≥ 90 mmHg for more than 2 hours. Subsequently, the BPMS was attached to the skin, allowing the microneedles to penetrate into the pig skin (Fig. 6I). After a 1-hour monitoring-feedback intervention with Cu-NC@PMN@Pt–coupled BPMS, the mean heart rate (HR) of the pigs decreased from 100.2 ± 6.7 to 68.2 ± 5.8 beats per minute (bpm). Meanwhile, the SBP decreased from 172.6 ± 7.5 to 119.8 ± 7.4 mmHg, and the DPB decreased from 106.4 ± 8.8 to 81.8 ± 9.2 mmHg (Fig. 6, J and K). In contrast, pigs treated with Pt@PMN-coupled BPMS still retained high blood pressure levels (SBP > 140 mmHg; DBP > 90 mmHg), as well as the control group (fig. S23). Overall, Cu-NC@PMN@Pt–coupled BPMS successfully regulates NO generation via electro-enzymatic cascade catalysis for in vivo vasodilation in a monitoring-feedback manner.

## DISCUSSION

In this study, we present a skin-integrated, wearable BPMS that enables closed-loop hypertension control through real-time monitoring and electro-enzymatic cascade–triggered NO delivery. The system integrates multiple functional modules into a flexible patch, offering both diagnostic and therapeutic capabilities in a closed-loop manner.

Accurate blood pressure sensing is the prerequisite for intelligent feedback regulation. Our reflection-mode PPG architecture, featuring dual LED/PD pairs and adaptive preprocessing algorithms, delivers stable blood pressure signals with reduced motion artifacts under mild motion conditions (fig. S5). Nonetheless, we acknowledge that large-amplitude movement (e.g., running and joint flexion) may compromise microneedle-tissue coupling and introduce signal distortion (*58*). To address this, future iterations will focus on (i) incorporating microneedles into stretchable substrates with serpentine interconnects to improve conformability, (ii) decreasing interface impedance artifacts and developing real-time contact impedance monitoring to detect interface instability, and (iii) integrating active motion compensation modules and adaptive filtering algorithms to enhance robustness under dynamic conditions.

In terms of therapeutic continuity, the microneedle electrode patch demonstrated a continuous NO release duration of ~6 hours from a single hydroxyurea loading (100 μl; 1 mM), with negligible performance loss across repeated cycles (fig. S24). This stability highlights the durability of both the catalytic nanomaterials and the PGMA-based PMN scaffold. The electrocatalytic layer, comprising in situ–deposited Au NPs encapsulated in agar gel and spatially confined within a hydroxyurea-loaded sponge, was structurally reinforced by a multilayered architecture. This includes rigid chamber housing, cohesive hydrogel interfaces, and overlying microneedle protection, together preserving catalyst integrity and minimizing leaching or degradation (fig. S25).

Power autonomy is another critical consideration for real-world deployment. Our estimated power budget (table S1) and battery discharge tests indicate a runtime of ~7 hours in idle mode and ~3 hours during active NO generation (work mode) with a 100–mA·hour lithium-ion battery (fig. S26). To further enhance operational endurance, we upgraded the battery to a higher-capacity 650–mA·hour lithium-ion cell. Experimental measurements with this configuration (fig. S27) demonstrated a significantly extended runtime of ~45 hours in idle mode and ~ 20 hours in work mode, enabling prolonged monitoring and therapeutic application cycles. In addition to scaling battery capacity, we are actively implementing further optimizations: (i) configuring the MCU for deep sleep and adaptive sensing intervals to reduce standby power, (ii) eliminating the OLED module and relying on Bluetooth-synced smartphone display to minimize active power consumption, and (iii) exploring wireless power delivery solutions such as near field communication (NFC) and Qi-standard inductive charging to support convenient and continuous operation in future wearable iterations.

While the current system was designed with modular architecture for testing and debugging, miniaturization was considered from the outset. A more compact prototype was also developed, integrating the pulse sensing chip, logic unit, electrodes, microneedles, and drug reservoir on a single flexible substrate (fig. S28). Future designs will further reduce footprint and enhance skin conformity by eliminating discrete battery and display modules, leveraging stretchable electronics, and optimizing adhesive architecture.

At the physiological level, this study validates the principle that localized NO release can induce systemic blood pressure reduction. Although NO has a short biological half-life (~10 s) and limited diffusion distance (~100 to 500 μm), its local delivery at the dermal microvascular interface leads to vasodilation of arterioles and capillaries. This reduces total peripheral resistance (TPR) and thereby lowers mean arterial pressure (MAP), following the relation: MAP = cardiac output × TPR. This mechanism parallels that of US Food and Drug Administration–approved nitroglycerin patches and recent transdermal sodium nitroprusside microneedle platforms, which lower systemic blood pressure through localized NO delivery (*24*, *59*–*61*). Our BPMS builds on this principle by enabling programmable, real-time NO generation and delivery through positively charged PMN, further augmented by EOF for directional transport. Moreover, our use of porcine skin as the in vivo model ensures high translational relevance. Pig skin closely resembles human skin in thickness, vascular structure, and barrier properties, making it a standard surrogate in transdermal research (*62*, *63*). Given the comparable NO flux achieved at the tissue interface, our findings provide a solid foundation for human application. While direct human trials were not conducted in this study, our microneedle patch is inherently modular and scalable. By increasing patch area, drug reservoir volume, or stimulation parameters, NO output can be adjusted without altering the underlying catalytic design. This positions our BPMS as a broadly applicable platform for precision cardiovascular therapy and future clinical translation.

## MATERIALS AND METHODS

### Fabrication of Au NPs on carbon cloth

The deposition of Au NPs was carried out in an electrochemical workstation (CHI760E, CH Instruments). The three-electrode system comprised a carbon cloth as the working electrode, a Ag/AgCl electrode as the reference electrode, and a Pt wire serving as the counter electrode. The electrolyte was prepared with a composition of EDTA (17 mM), K_2_HPO_4_ (170 mM), Na_2_SO_3_ (1.27 M), and HAuCl_4_•3H_2_O (3 mM). Au NPs were fabricated on the carbon cloth through an electrochemical deposition process, monitored by a current-time (*I*-*t*) curve, at a constant potential of −0.8 V for 10 min. Afterward, the samples were thoroughly rinsed with water and dried under a stream of nitrogen gas, resulting in the formation of Au NPs on the carbon cloth.

### Preparation of Cu-NC NBs

Cu-NC NBs were prepared by solvothermal method. Initially, Cu(CH_3_COO)_2_•H_2_O (0.6 g) and urea (1 g) were dissolved in ethanol (30 ml), followed by the addition of PEI (5 ml; 0.1 g ml^−1^) solution in ethanol. The mixture was transferred to a 50-ml Teflon-lined autoclave and heated at 180°C for 12 hours. The resulting precipitate was washed three times by centrifugation (10,000*g* for 5 min) with water and subsequently dispersed in water for further use.

### Fabrication of PMN array

A monomer stock solution was prepared by combining glycidyl methacrylate (1 ml), trimethylolpropane trimethacrylate (0.52 ml), and triethylene glycol dimethacrylate (1.57 ml). A porogen stock solution was prepared by dissolving polyethylene glycol (0.4 g; 8 kDa) in ethylene glycol methyl ether (2 g) at 50°C. Subsequently, the monomer and porogen stock solutions were mixed in a 6:7 volume ratio, followed by the addition of Irgacure 184 (1 wt % of the monomer). The mixture was poured into a polydimethylsiloxane mold (Taizhou Microchip Medical Technology Co., Ltd.) with a needle length of 2000 μm and a spacing of 1.2 mm on a planar substrate (ϕ10 mm by 1 mm in thickness). The mold underwent vacuum extraction for 10 min to eliminate air bubbles, and photopolymerization was conducted under 365-nm ultraviolet (UV) light for 20 min. PMNs were obtained by removing the porogen with an ethanol/water mixture (1:1, v/v) at 60°C for 12 hours.

### Synthesis of Cu-NC@PMN

Cu-NC NBs (1 mg) were dispersed in the mixture (10 ml) of the monomer and porogen stock solutions. The Cu-NC@PMNs were prepared following the above described procedures.

### Synthesis of Cu-NC@PMN@Pt

The synthesis of Cu-NC@PMN@Pt was conducted by first immersing Cu-NC@PMN (ϕ10 mm by 1 mm in thickness) in a PEI solution (10 ml; 10 mg ml^−1^) for 20 min. Subsequently, the material was incubated in a K_2_PtCl_4_ solution (10 mM) at 60°C for 5 min, followed by the addition of NaBH_4_ aqueous solution (500 μl; 60 mg ml^−1^). After 1 hour, the obtained Cu-NC@PMN@Pt was washed with an ethanol/water mixture (1:1, v/v) at room temperature for 24 hours to remove the pore-forming agent.

### Microneedle electrode patch assembly

First, the Ag/AgCl paste was coated onto the FPCB anode by masking method and allowed to dry using a hot air blower. Subsequently, the 3D-printed PLA cathode chamber (a cylinder hollowed out at both ends, with a diameter of 1 cm and a wall thickness of 2 mm) was fixed to the FPCB cathode. Next, the Au NP electrocatalysts deposited on carbon cloth were encapsulated in agar gel and sandwiched within a spatially matched buffer sponge preloaded with 100 μl of 1 mM hydroxyurea and then placed into the cathode chamber. The microneedle arrays were placed and fixed on the cathode chamber and encapsulated with double-sided 3M medical tape. Last, a conductive hydrogel (agar gel containing 0.9% NaCl) was loaded on the Ag/AgCl anode.

### Fabrication of flexible circuit

The custom-designed flexible board mainly consists of passive components (capacitors, resistors, and diodes), a pulse sensor (YK1801, Yunkear Technology), a signal processing chip (HR6707, Yunkear Technology), a gain chip (HR6816, Yunkear Technology), an algorithmic chip (SFB9712, Yunkear Technology), a programmable low-power microcontroller (ESP32-U4WDH, Espressif), an antenna for wireless communication, a crystal oscillator (40 MHz; Jingyou Electronic), a rechargeable 100–mA·hour lithium-ion battery, two low-dropout regulators (LDOs; BL919833BA, Belling) for voltage conversion, and an OLED display. All circuit components were hot-air blown with tin-lead solder paste.

### H_2_O_2_ generation/detection

H_2_O_2_ quantification was accomplished using Amplex Red as a sensor, which yields an intense red fluorescent product (λ_ex_ = 530 nm; λ_em_ = 590 nm) in the presence of HRP. Subsequently, the H_2_O_2_ concentration was measured using a fluorescence spectrophotometer (F-4600, Hitachi, Japan).

In the three-electrode system, electrocatalytic generation of H_2_O_2_ was performed via constant potential electrolysis in an H-cell. A carbon cloth deposited with Au NPs served as the working electrode, while a Pt sheet and a Ag/AgCl electrode were used as the counter and reference electrode, respectively. The chambers were separated by a proton exchange membrane (Nafion 117, DuPont), and the working electrode chamber was filled with Amplex Red (6 ml; 25 μg ml^−1^) and HRP (100 μg ml^−1^) in PBS (pH 7.4; 0.01 M). Then, samples (200 μl) were collected every 5 min under a constant potential of −0.3 V (versus Ag/AgCl) for fluorescence analysis.

For the two-electrode system powered by DC (JDS6600, Cleqee), the carbon cloth with Au NPs acted as the cathode. A carbon cloth coated with Ag/AgCl paste (0.1 mg cm^−2^; JLL10, Julonghuina, Shanghai) served as the anode. The electrolytic cell was filled with PBS (6 ml; pH 7.4; 0.01 M) containing Amplex Red (25 μg ml^−1^) and HRP (100 μg ml^−1^). After 30 min of electrolysis at various voltages (0.2, 0.4, 0.6, 1.0, and 3.0 V), H_2_O_2_ production was assessed on the basis of the solution fluorescence analysis.

In the biological tissue system, the cathode and anode were embedded within an agarose hydrogel (2% w/v in 0.01 M PBS) containing Amplex Red (25 μg ml^−1^) and HRP (100 μg ml^−1^). These electrodes were separated by a section of pork tissue with dimensions of 10 cm by 5 cm by 2 cm (*l* by *w* by *h*). H_2_O_2_ generation was then assessed after 30 min of electrolysis at the aforementioned voltages, using the above-described method.

### NO generation/detection

NO generation was quantified using DAF-FM as a fluorescent probe. DAF-FM was synthesized from DAF-FM DA via hydrolysis in NaOH aqueous solution. Specifically, DAF-FM DA (2 μl; 5 mM) in dimethyl sulfoxide was added into NaOH solution (80 μl; 10 mM). After 1 hour of hydrolysis, PBS (918 μl; pH 7.4; 10 mM) was added to yield the DAF-FM solution (20 μM).

For NO detection, hydroxyurea served as the substrate in a cascade reaction catalyzed by Au NPs and HRP. In a two-electrode system powered by DC (JDS6600, Cleqee), a carbon cloth decorated with Au NPs functioned as the cathode, while a carbon cloth coated with Ag/AgCl paste (0.1 mg cm^−2^; JLL10, Julonghuina, Shanghai) acted as the anode. The electrolytic cell was filled with PBS (6 ml). Electrolysis was performed at different voltages (0.2, 0.4, 0.6, 1.0, and 3.0 V) for 30 min. Electrolyte (100 μl) was taken out and incubated with assay solution [100 μl; containing DAF-FM (2 μM), hydroxyurea (1 mM), and HRP (100 μg ml^−1^)] for 20 min. NO production was evaluated on the basis of the DAF-FM fluorescence (λ_ex_ = 470 nm; λ_em_ = 525 nm).

In the biological tissue system, the cathode and anode were embedded in an agarose hydrogel (2% w/v in 0.01 M PBS). These electrodes were separated by a section of pork tissue with dimensions of 10 cm by 5 cm by 2 cm (*l* by *w* by *h*). NO production was then assessed after 30 min of electrolysis at various voltages (0.2, 0.4, 0.6, 1.0, and 3.0 V) using the described method above.

### CV measurements

CV experiments were conducted using an electrochemical workstation (CHI760E, CH Instruments). A carbon cloth substrate with electrodeposited Au NPs served as the working electrode, paired with a Ag/AgCl reference electrode and a Pt sheet as the counter electrode. The measurements were performed in a PBS solution (0.01 M; pH 7.4) with a scan rate of 100 mV s^−1^ at room temperature.

### Evaluation of EOF through water transport

EOF assessment was conducted using a modified positively charged porous substrate (ϕ20 mm by 2.0 mm). This substrate was integrated into an H-type closed electrolytic cell (Dongsilica Quartz, China) equipped with a horizontal capillary (inner diameter of 1.5 mm). A constant DC voltage ranging from 0 to 3 V, supplied by a signal generator (JDS6600, Cleqee), was applied to determine the EOF strength of the PMN. PBS (10 mM; pH 7.4) served as the electrolyte solution. Ag/AgCl filament (ϕ2.5 mm by 10 cm) was used as cathode (AgCl + e^−^ → Ag + Cl^−^), and Ag filament (ϕ2.5 mm by 10 cm) was used as anode (Ag^+^ + e^−^ → Ag) to prevent bubble generation. The EOF rate was quantified by measuring the displacement of the water surface within the horizontal capillary.

### NO transdermal delivery in skin

To investigate the potential of EOF to promote NO transdermal delivery, a piece of pig skin [4 cm by 4 cm by 0.5 cm (*l* by *w* by *h*)] was pretreated by incubating with the NO probe for 6 hours under dark conditions. The assembled microneedle electrode patch was applied to the porcine skin and connected to a DC power supply (1 V) for 30 min. The patches were then removed, and the skin was sectioned into slices of 20-μm thickness using a cryosectioner (CM1850-1-1, Leica, Germany). These sections were examined with a laser confocal microscope (FV3000, Olympus, Japan) to assess the NO penetration. Fluorescence images were analyzed using ImageJ software, applying background subtraction and intensity thresholding to determine the effective NO delivery area. The transdermal delivery area (in square millimeters) was quantified by integrating the fluorescence-positive pixels over the scanned area, and vertical penetration profiles were obtained along the *z* axis.

### Test of cytocompatibility

HUVECs were cultured in Dulbecco’s modified Eagle’s medium (DMEM) at 5% CO_2_ atmosphere. PMN (5 g) was immersed in DMEM (50 ml) for 24 hours to provide PMN leachate (0.1 g ml^−1^). HUVEC cells were seeded into 96-well plates at the density of 1 × 10^4^ cells per well. After 12 hours of incubation, cells were incubated with culture medium containing leachate for 24 hours. Cell viability was measured using cholecystokinin-8 assay kit following the manufacturer’s protocols. The cells were stained with propidium iodide (PI; 5 μM) and calcein AM (5 μM) for 20 min and then imaged with an inverted fluorescence microscope (IX83, Olympus, Japan).

The Au NP cathode was assembled on PMN through a water-containing sponge. The PMNs were inserted into porcine skin and connected to a DC power supply (1 V) to initiate the electrocatalytic ORR. H_2_O_2_ levels in tissue were assessed by DHE, which undergoes oxidative dehydrogenation and binds to DNA to produce red fluorescence. After 30 min, the skin was sectioned into 20-μm-thick slices using a cryosection machine (CM1850-1-1, Leica, Germany), which were then imaged under a laser confocal microscope (FV3000, Olympus, Japan) for analyzing the ROS levels.

### BPMS-mediated vasodilation in vitro

Isometric aortic tonometry was conducted using an in vitro microvascular tonometry system (DMT 620M, Denmark). After the administration of anesthesia to New Zealand White rabbits, the thoracic aorta was swiftly excised. The connective tissue was then removed, and the aorta was sectioned into 5-mm rings (five rings per experimental group, derived from five rabbits). Each ring was secured at one end to a stationary hook and at the opposite end to a force transducer. The experiments were carried out in individual chambers, each containing a bath with Krebs-Henseleit solution [8 ml; comprising 118 mM NaCl, 4.7 mM KCl, 2.4 mM CaCl_2_, 1.2 mM KH_2_PO_4_, 2.4 mM MgSO_4_, 25 mM NaHCO_3_, and 11.1 mM glucose (pH 7.4)], and maintained under physiological oxygen conditions at 37°C. After 30 min of equilibration, KCl solution (60 mM) was added via syringe. When the tension reached equilibrium, the microneedle electrode was inserted into the surface of the rabbit skin, and the other side of the rabbit skin was in contact with the Krebs-Henseleit solution. Electrocatalysis was conducted at a DC voltage of 1 V. Changes in tension (ΔTension) and relaxation (%) were calculated as follows

$$\left(\text{ΔTension}\right)=F_{0}–F_{t}$$ (1)

$$\text{Relaxation}\;\left(\%\right)=\left(F_{0}–F_{t}\right)/F_{0}\times 100\%$$ (2)

In these equations, *F*_0_ and *F*_t_ represent the aortic tension measured before and after treatment with BPMS, respectively.

### BPMS-mediated NO generation and vasodilation in vivo

New Zealand White rabbits (*n* = 5 per group) received a 30-min administration of BPMS to induce NO production. Subsequently, the rabbits were euthanized, and their thoracic arteries were harvested. A third of these specimens were preserved in 10% neutral buffered formalin, embedded in paraffin, sectioned into 5-μm slices, and stained with H&E for histological examination. Morphometric analysis was performed using an inverted microscope (IX83, Olympus, Japan). The remaining arterial segments were incubated with the NO-specific intracellular fluorescent probe DAF-FM DA (2 μM) for 20 min in darkness. The thoracic arteries were then cleansed, divided into two groups at random, and further processed. The first group was subjected to fluorescence imaging using an in vivo imaging system (IVIS Lumina III, USA). The second group underwent cryosectioning, and 5-μm arterial sections were prepared using a cryosectioner (CM1850-1-1, Leica, Germany), mounted on slides, and stained with 4′,6-diamidino-2-phenylindole (DAPI). After PBS rinsing, these sections were visualized under a laser scanning confocal microscope (FV3000, Olympus, Japan).

Hemodynamic parameters of the ventral artery of New Zealand White rabbits (*n* = 5 per group) were assessed by spectral Doppler ultrasonography (Resona R9, Mindray, China). Briefly, sodium pentobarbital (8 ml; 1%) was injected intravenously via the ear margin, followed by a 30-min BPMS treatment. The abdominal aorta was imaged by ultrasonography to analyze blood flow velocities and calculate the RI.

Guangxi Bama pigs (*n* = 5 per group) received intramuscular injection of metaraminol bitartrate at a dosage of 0.1 mg kg^−1^. When the blood pressure is maintained at SBP ≥ 140 mmHg and DBP ≥ 90 mmHg for more than 2 hours, hypertensive pig model was successfully constructed. Subsequently, the pigs received a 1-hour monitoring-feedback intervention of BPMS. During the treatment, HR and blood pressure data were collected and analyzed.

### Cell lines and animals

HUVECs were obtained from Xiangya Hospital, Central South University (Changsha, China). The animal experiments were approved by the Institutional Animal Care and Use Committee, the Second Xiangya Hospital of Central South University (no. 20220286). All animal experiments are performed in accordance with the regulations of the Laboratory Animal Center of Xiangya School of Medicine, Central South University.

### Ethics declarations

This human participant study was approved by the Ethics Committee for Clinical Research of the Second Xiangya Hospital, Central South University (LYEC2025-0064). All participants provided written informed consent after receiving full disclosure of the study’s purpose, procedures, risks, benefits, and their rights to voluntary participation and withdrawal.

### Statistical analysis

All data were statistically analyzed using one-way analysis of variance (ANOVA) with Tukey’s post hoc test and are expressed as means ± SD, with *P < 0.05, **P < 0.01, ***P < 0.001, and ****P < 0.0001. ns denotes no significant difference (*P* > 0.05).
