# Supplementary material for: A closed-loop bioelectronic patch for intelligent blood pressure management
Source: Sci Adv. 2025 Aug 6;11(32):eadx6438. doi: 10.1126/sciadv.adx6438 (PMC12327451; doi:10.1126/sciadv.adx6438)
Supplement: Supplementary file 1 — Supplementary Text Figs. S1 to S28 Table S1 [file sciadv.adx6438_sm.pdf]

Science Advances
AAAS

Supplementary Materials for

A closed-loop bioelectronic patch for intelligent blood pressure management

Yuyan Zou *et al.*

Corresponding author: Wansong Chen, chenws@csu.edu.cn; You-Nian Liu, liuyounian@csu.edu.cn; Yanli Zhao, zhaoyanli@ntu.edu.sg

Sci. Adv. **11**, eadx6438 (2025)
DOI: 10.1126/sciadv.adx6438

This PDF file includes:

Supplementary Text
Figs. S1 to S28
Table S1

Supplementary Text

Materials and reagents

Copper (II) acetate hydrate, dipotassium phosphate (K_2HPO_4), ethylene diamine tetracetic acid (EDTA), hydrogen peroxide (H_2O_2, 30%), polyethyleneimine (PEI, MW=10000), sodium borohydride ($NaBH_4$), sodium sulfite (Na_2SO_3), and urea were purchased from Sinopharm Group Chemical Reagent Co., LTD. 4-Amino-5-methylamino-2',7'-difluorofluorescein diacetate (DAF-FM DA) probe, Amplex red, horseradish peroxidase (HRP), and WST-8 assay kit were obtained by Shanghai Beyotime Institute of Biotechnology. Chloroauric acid ($HAuCl_4 \cdot 3H_2O$) and potassium tetrachloroplatinate (K_2PtCl_4) were provided by Shanghai Haohong Biomedical Technology Co., LTD. Ethylene glycol methyl, glycidyl methacrylate, Irgacure 184, polyethylene glycol (8 kDa), triethylene glycol dimethacrylate, and trimethylolpropane trimethacrylate were purchased from Shanghai Merrier Biochemical Technology Co., LTD. Calcein acetoxymethyl (calcein-AM), CCK-8 cell viability assay kit, and propidium iodide (PI) were provided by Shanghai Hongye Biotechnology Co., LTD.

Characterization

The morphology and elemental mapping of Cu-NC NBs were observed on a transmission electron microscope (TEM; JEOL, JEM-2100F, 200 kV). Morphology and surface structures of porous microneedle (PMN) arrays were observed by scanning electron microscopy (FE-SEM; JSM-7610FPlus, JEOL, Japan). The chemical composition of the microneedle surface was determined by X-ray photoelectron spectroscopy (XPS, K-Alpha+, ThermoFisher Scientific, USA) and Fourier transform infrared (FTIR, Nicolet iS50, ThermoFisher Scientific, USA) spectroscopy. The mechanical properties and pore size distribution of the microneedles were determined using a universal testing machine (5543A, Instron, USA) and a fully automated piezomercury pore size analyzer (AutoPore IV, Micromeritics, USA), respectively. UV-visible absorption spectra and fluorescence spectra were measured on a spectrophotometer (UV-2450, Shimadzu, Japan) and (F-4600, Hitachi, Japan), respectively. X-ray diffraction (XRD) analysis was performed on an X-ray autodiffractometer (SIMENS D500, Switzerland). Cell fluorescence imaging was observed by an inverted fluorescence microscope (IX-83, Olympus, Japan).

Peroxidase (POD)-mimic activity

The POD-mimic activity of Cu-NC NBs was assessed using TMB as a substrate. A working solution was prepared by combining TMB (50 μL, 20 mM) and H_2O_2 (50 μL, 10 mM) in PBS (1 mL, pH 7.4, 10 mM). Then, the working solution (900 μL) was incubated with Cu-NC NBs (100 μL, 100 μg mL^{-1}) for 10 min. The supernatant was subjected to UV-vis absorption spectroscopy (UV-2450, Shimadzu, Japan) to determine the peroxidase-mimic activity.

To quantify the POD-mimic activity, H_2O_2 (10 μL, 0, 1, 2, 5, 10, 20, and 50 mM) aqueous solution, TMB solution (10 μL, 20 mM) in DMSO, and Cu-NC NBs (10 μL, 200 μg mL^{-1}) in water were added into PBS buffer (170 μL, 10 mM, pH 7.4). The oxidation of TMB was monitored over 10 min at an absorbance of 652 nm. The reaction rate (v) was calculated from the initial slope of the TMB oxidation curve. The Michaelis-Menten constants (K_M and V_{max}) were calculated by fitting the data to the Michaelis-Menten saturation curve:

$$v = \frac{c(H_2O_2) \times V_{max}}{c(H_2O_2) + K_M}$$

Catalase (CAT)-mimic activity

O_2 evolution from the catalytic decomposition of H_2O_2 was monitored using an oxygen dissolver (JPB-607A, Leici, China). A volume (100 μL) of Pt NPs (100 μg mL^{-1}) or Pt@PMN (1 mg, containing Pt 1 wt%) was added into H_2O_2 (10 mL, 0.1 M).

To quantify the CAT-mimic activity of Pt NPs, the Pt NP solution (2 mL, 20 μg mL^{-1}) was mixed with H_2O_2 (2 mL) at different concentrations (0, 0.02, 0.04, 0.1, 0.2, and 0.4 M) in PBS buffer (10 mM, pH 7.4). O_2 production was measured using a dissolved oxygen meter (JPB-607A, Leici, China). K_M and V_{max} were calculated from the Michaelis-Menten saturation equation.

Superoxide dismutase (SOD)-mimic activity

The SOD-mimic activity was assessed using WST-8 as a probe, with SOD (100 U mL^{-1}) serving as a positive control. Initially, a working solution was prepared by combining WST-8 (950 μL, 1 mM) and xanthine oxidase (50 μL, 100 U mL^{-1}). Subsequently, Pt NPs (100 μL, 100 μg mL^{-1}), Pt@PMN (1 mg, containing Pt 1 wt%) or SOD (100 μL, 100 U mL^{-1}) aqueous solution was added into the working solution (800 μL). The mixture was incubated with xanthine (100 μL, 1 mM) at 37 °C for 30 min. The supernatant was subjected to UV-visible spectroscopy (UV-2450, Shimadzu, Japan) to measure the absorption spectrum.

To quantify SOD-mimic activity of Pt NPs, a total SOD assay kit (Beyotime, China) was utilized following to the manufacturer's protocols. The superoxide radical was generated by the oxidation of hypoxanthine by xanthine oxidase, reducing WST-8 into a yellow formazan. In the presence of Pt NPs with SOD-mimic activity, the formazan generation was competitively inhibited. A range of final concentrations (0, 1, 2, 3, 5, 10, 20, 40, and 80 μg mL^{-1}) of Pt NPs was tested to determine the inhibition rate of the WST-8 reduction. Inhibition rate was calculated as follows:

$$\text{Inhibition rate (\%)} = \frac{\Delta A_{blank} - \Delta A_{assay}}{\Delta A_{blank}} \times 100\%$$

In the equation, ΔA_{blank} represents the absorbance change of the blank control, and ΔA_{assay} is the absorbance change of the assay samples.

Definition of SOD activity unit: In the xanthine oxidase coupled reaction system described above, an inhibition of 50% is equivalent to one unit of enzyme activity. The SOD activity in the reaction system was calculated as follows:

$$\text{SOD activity (U/mg)} = \frac{\text{Inhibition rate}}{\omega \times (1 - \text{Inhibition rate})}$$

In the equation, ω denotes the mass of Pt NPs (mg) used in the reaction system at a specific inhibition rate.

Finite element simulation

EOF simulation and substance concentration simulation were conducted using the electrochemical and fluid modules of the COMSOL Multiphysics software. In the first part of the model, the continuity equations for steady state velocity and current density were solved as:

$$\nabla \cdot \boldsymbol{u} = 0$$
$$\nabla \cdot \boldsymbol{i} = 0$$

where $\boldsymbol{u}$ represents velocity (m s^{-1}) and **i** denotes the current density vector (A m^{-2}). The velocity is driven by two driving forces including the pressure gradient and the electroosmotic force.

$$\boldsymbol{u} = -\frac{\varepsilon_P a^2}{8\mu\tau}\nabla p + \frac{\varepsilon_P \varepsilon_W \zeta}{\mu\tau}\nabla V$$

In this equation, ε_P denotes the porosity, a is the mean radius of the pores (m), μ denotes the fluid's dynamic viscosity (Pas), τ denotes the tortuosity of the porous structure, ε_W is the fluid's dielectric constant (F m^{-1}), p denotes the pressure (Pa), ζ is the zeta potential (V), and V represents the electric potential (V). The equation is solved through the interface "partial differential equations in general form". The current density is modeled through the "Current" interface, which solves the following equation:

$$\boldsymbol{i} = -\kappa \nabla V$$

where κ denotes the conductivity (S m^{-1}). At the solid wall, the normal velocity component vanishes:

$$\boldsymbol{u} \cdot \boldsymbol{n} = 0$$

The boundary conditions for current density equilibrium are insulating for all boundaries except the electrode surfaces (where the potential is fixed):

$$\boldsymbol{i} \cdot \boldsymbol{n} = 0$$

$$V_{Cathode} = V_{\mathrm{Au}}$$

$$V_{anode} = V_{\mathrm{Ag/AgCl}}$$

In the second modeling stage, steady-state velocity and potential fields are used in the transient simulation of the charged product concentration of the chemical reaction system, assuming that the reaction products do not affect the conductivity or set potential in the porous structure. The mass transport equations for the products are solved through the "dilute matter transfer" interface as follows:

$$\frac{\partial_c}{\partial_t} + \nabla \cdot \boldsymbol{N} = 0$$

where $\boldsymbol{N}$ is the flux vector given by the Nernst-Planck equation:

$$\boldsymbol{N} = -D\nabla c - z u_m F c \nabla V + c\boldsymbol{u}$$

In this equation, D denotes the diffusion coefficient of the product (m^2 s^{-1}), c denotes its concentration (mol m^{-3}), z denotes the charge of the product, and F is the Faraday constant (C mol^{-1}). The mobility u_m (mol m^2 J^{-1} s^{-1})) is given by the Nernst-Einstein equation:

$$u_m = \frac{D}{R_{\mathrm{g}} T}$$

where R_{g} = 8.314 J mol^{-1} K^{-1}) is the gas constant and T (K) is the temperature.

Supplementary Figures

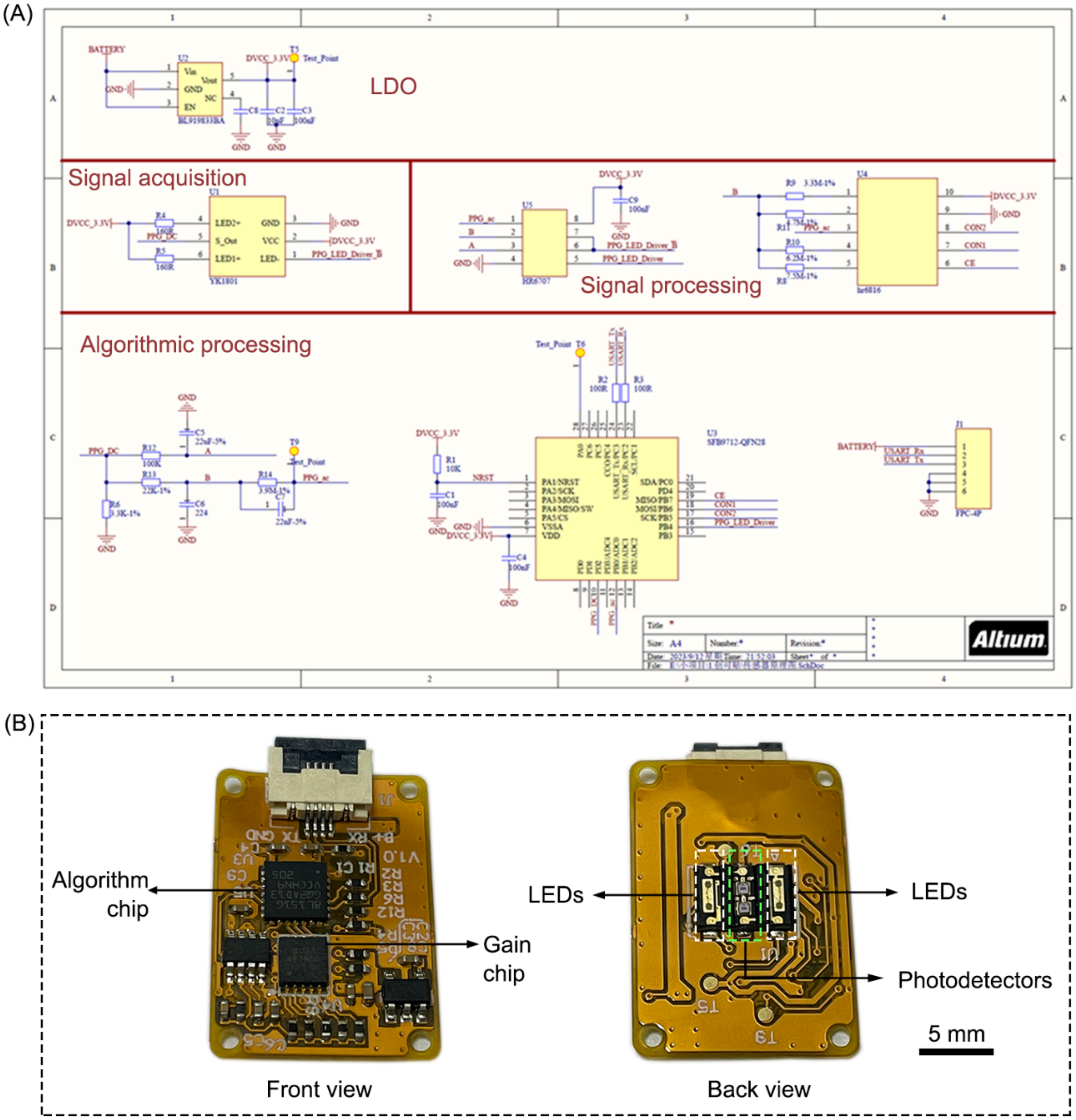

Fig. S1. Blood pressure sensing circuit diagram and actual images. (A) Circuit diagram for the design of the pulse sensing system. (B) Optical image of the pulse sensing system.

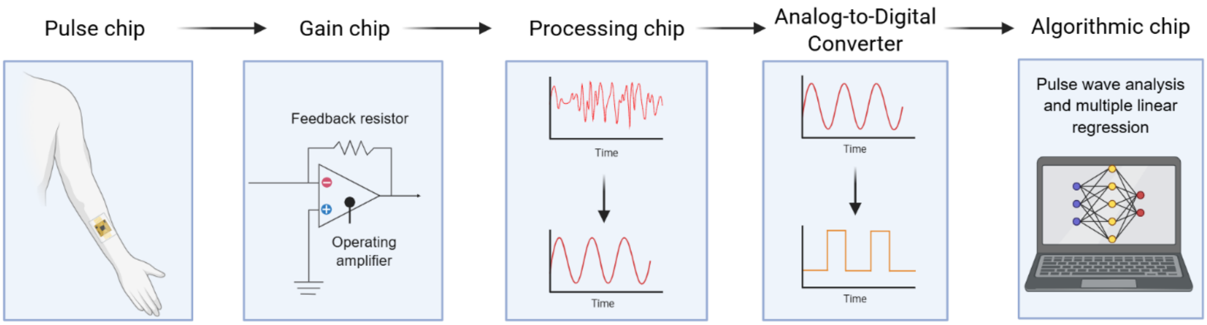

Fig. S2. A schematic flowchart of PPG-based signal processing for blood pressure estimation. The pulse chip captures optical signals modulated by pulsatile blood flow, which are then amplified by a low-noise gain chip to improve signal-to-noise ratio. The amplified signal is conditioned by a processing chip through baseline correction, band-pass filtering, and analog optimization. The cleaned analog signal is subsequently digitized by ADC, enabling low-power, periodic pulse waveform sampling. Finally, an algorithmic chip performs pulse wave analysis and applies a multiple linear regression model to estimate systolic and diastolic blood pressure in real time. This schematic was drawn by Biorender.com with permission.

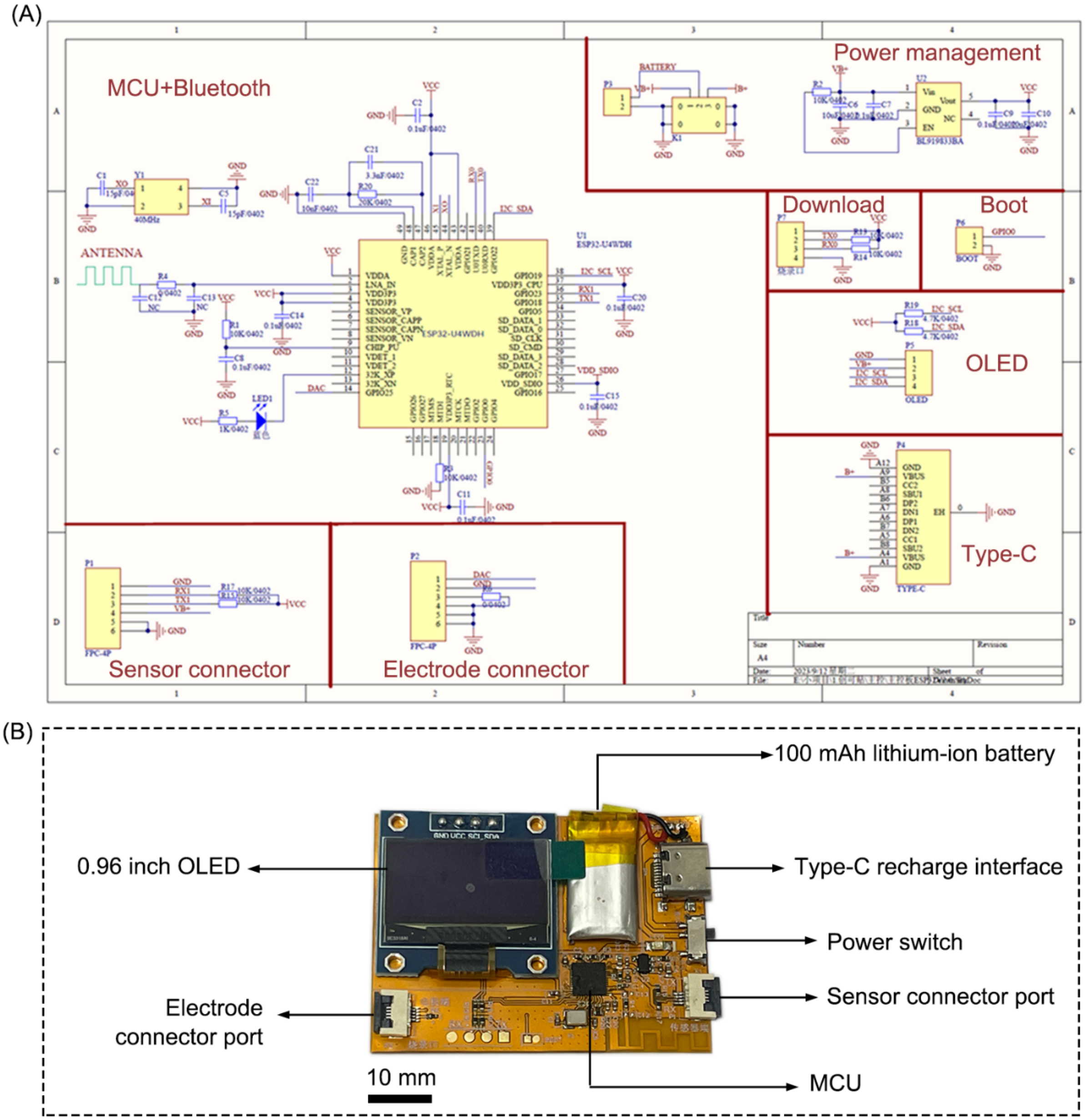

Fig. S3. Logic control circuit diagram and actual images. (A) Circuit diagram of the data logic control and transmission system. (B) Optical image of the data logic control and transmission system.

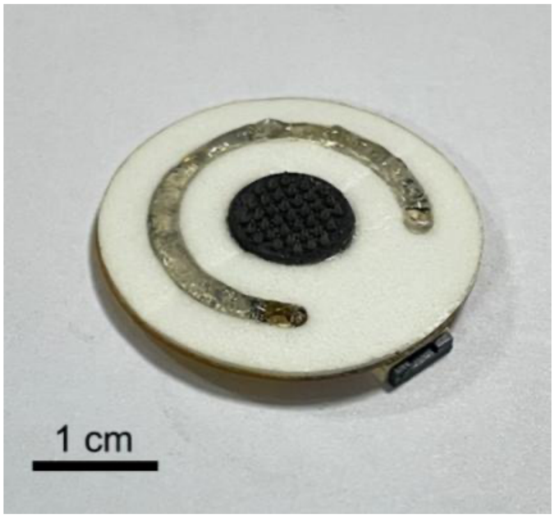

Fig. S4. **Top-view optical image of the assembled electrode system.**

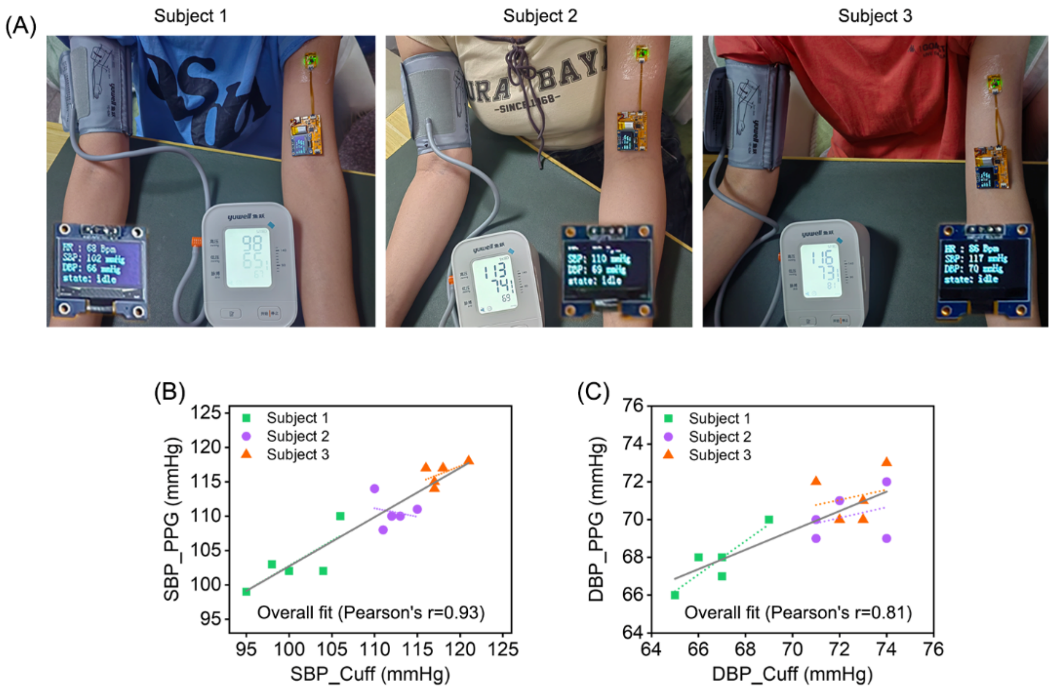

Fig. S5. Validation of PPG-based blood pressure monitoring against cuff-based measurements. (A) Photographs of three representative subjects wearing the smart patch on the forearm while undergoing simultaneous blood pressure measurement using a commercial cuff-based device (Yuwell S63AR, China) for reference. The OLED display shows real-time readings from the PPG system. (B) Correlation analysis between PPG-derived and cuff-based systolic blood pressure (SBP) and (C) diastolic blood pressure (DBP) for each subject (Subject 1: green squares; Subject 2: purple circles; Subject 3: orange triangles). Dotted lines indicate subject-specific linear fits, and the gray solid line represents the overall fit. Strong Pearson correlation coefficients were obtained across all subjects (r = 0.93 for SBP, r = 0.81 for DBP), validating the accuracy of the PPG-based system.

(A)
(B)
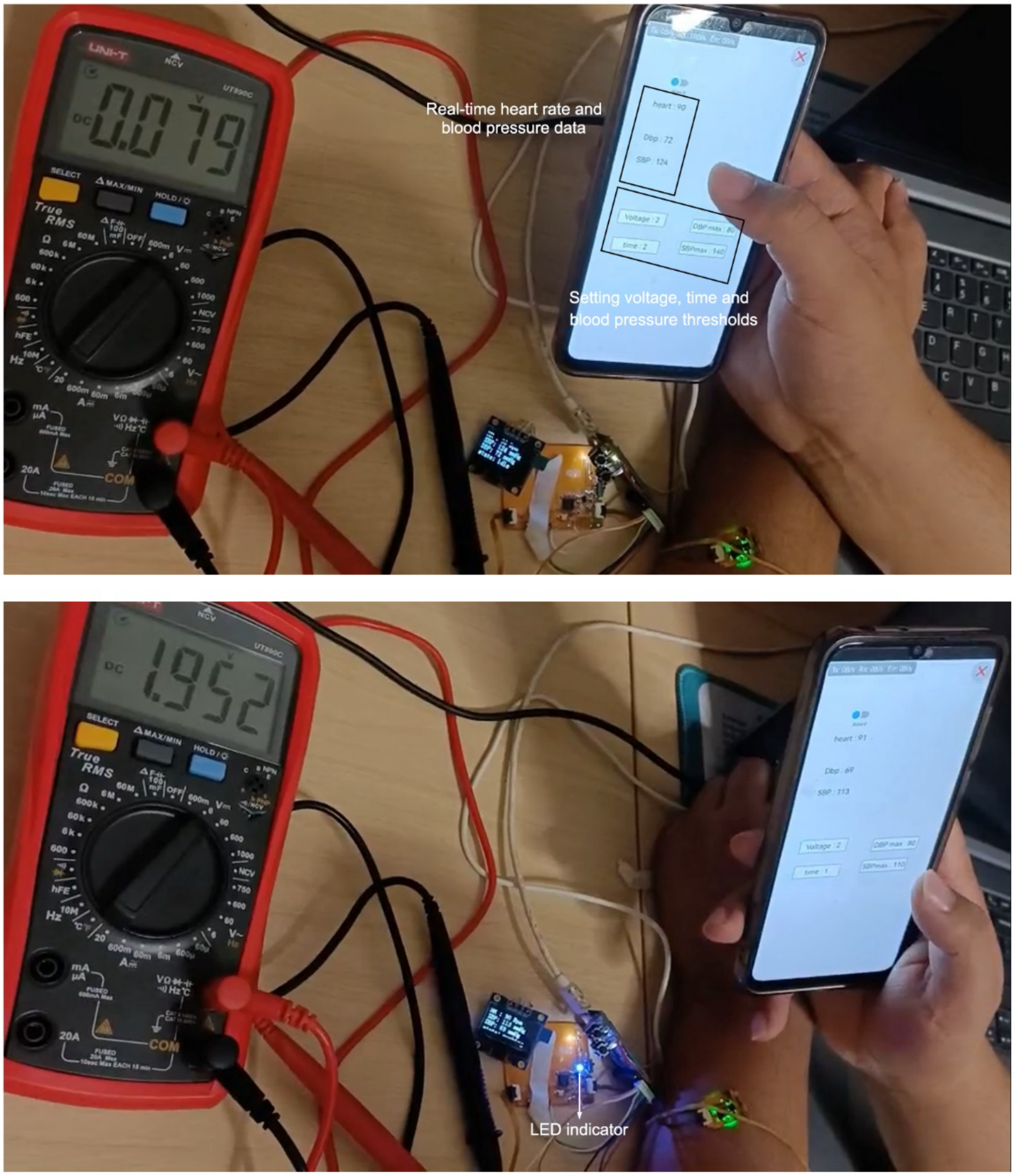

Fig. S6. **Validation of blood pressure responsiveness of BPMS.** The blood pressure was set to 140 mmHg systolic and 80 mmHg diastolic. The response voltage was set to 2 V with 2 min of response time. The tester wore the pulse sensing system, the real-time blood pressure value was less than the set blood pressure threshold, and the detected voltage at the cathode and anode terminals is almost 0. Thus, the response voltage doesn't work when the blood pressure of the subject is at the normal level (as shown in A). When the set systolic blood pressure was changed to 110 mmHg, the subject's systolic blood pressure was greater than the set blood pressure threshold, and the voltage detected at the cathode and anode terminals was nearly 2 V, and the working LED lighted up (as shown in B). Therefore, the BPMS displays a sensitive blood pressure response.

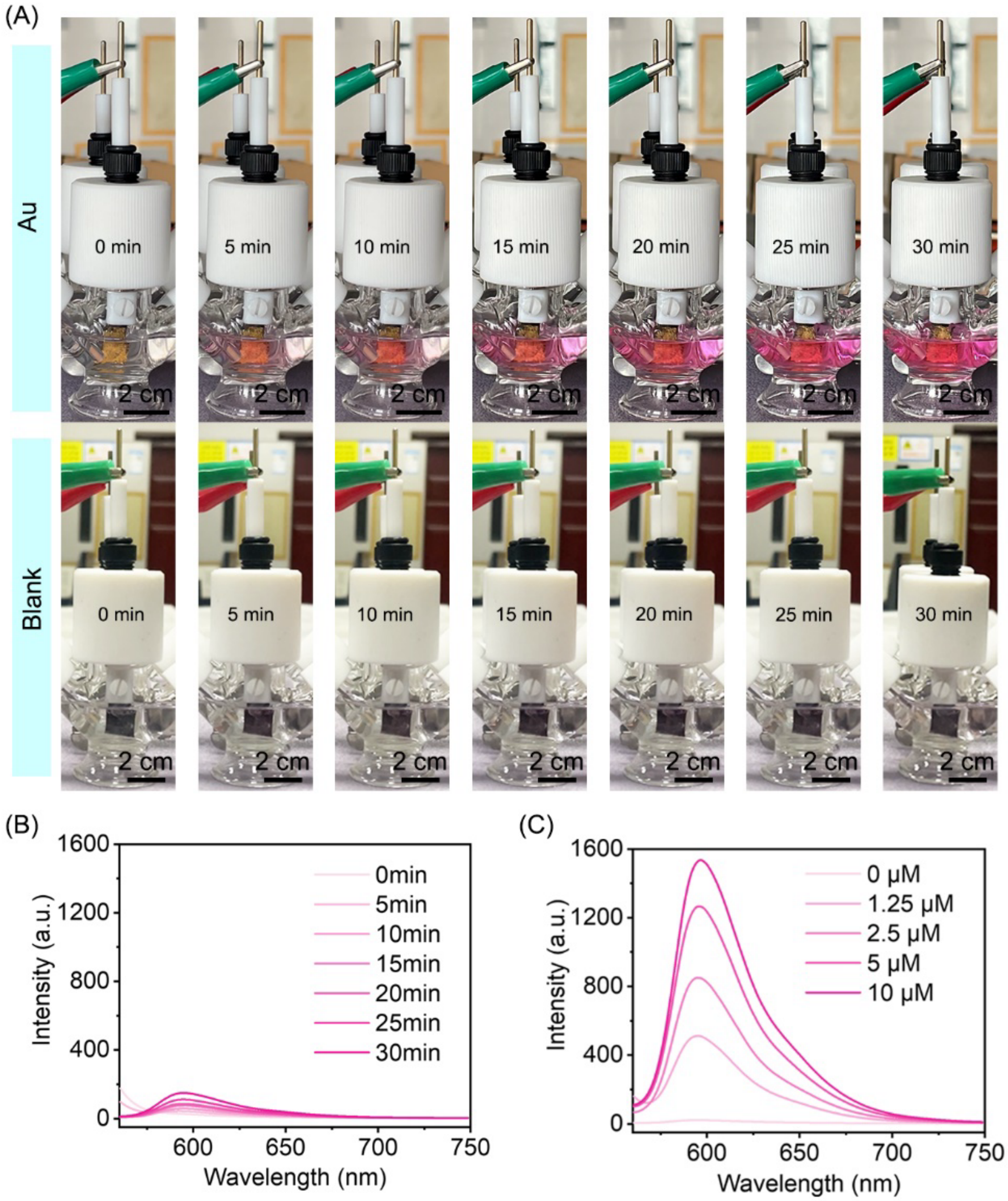

Fig. S7. Evaluation of electrogenesis of H_2O_2 by Au NPs. (A) Photographs of H_2O_2 production at different electrolysis times under the constant potential electrolysis at −0.3 V (vs. Ag/AgCl). (B) Fluorescence elevation of Amplex red indicates H_2O_2 generation from bare carbon cloth at −0.3 V (vs. Ag/AgCl) along with the electrocatalysis time. (C) Fluorescence spectra of Amplex red at different H_2O_2 concentration.

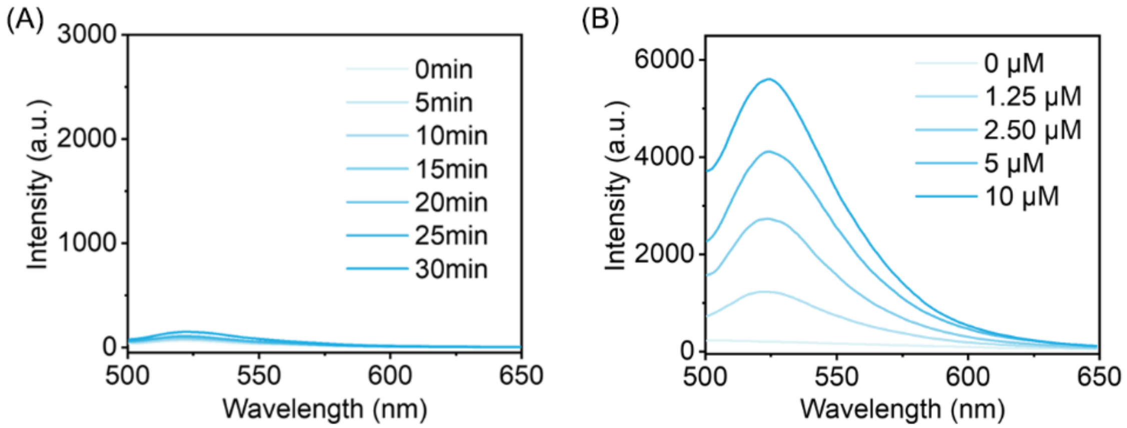

Fig. S8. Bare carbon cloth cascade generation NO assessment and NO standard curve. (A) Fluorescence spectra of DAF-FM indicate the cascade NO generation from bare carbon cloth under the constant potential electrolysis at −0.3 V (vs. Ag/AgCl) along with time. (B) Fluorescence spectra of DAF-FM at different NO concentration.

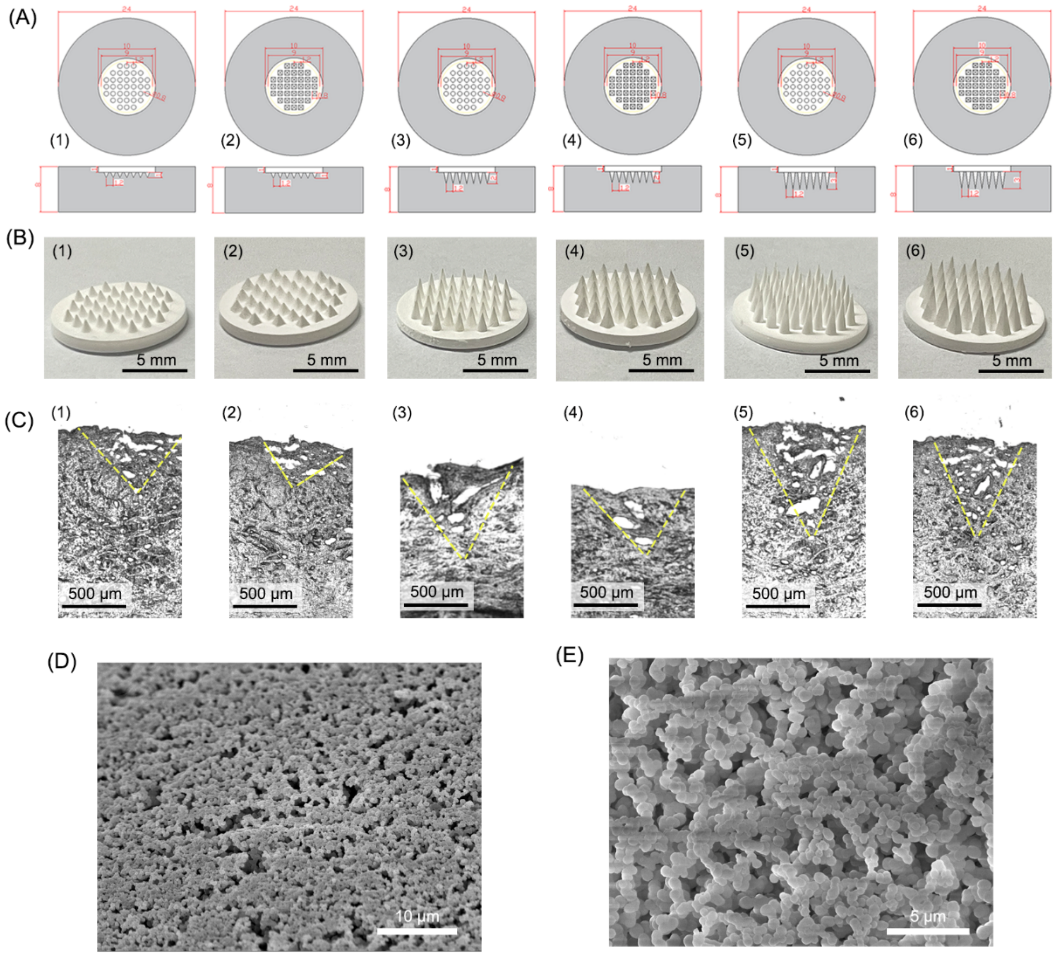

Fig. S9. Systematic structural optimization of porous microneedles in shape and length. (A) Schematic designs of six porous microneedle array configurations with different tip geometries and heights. Designs (1), (3), and (5) are conical microneedles with heights of 1 mm, 2 mm, and 3 mm, respectively. Designs (2), (4), and (6) are quadrangular pyramidal microneedles with the same respective heights. (B) Optical images of the corresponding fabricated porous microneedle arrays. All samples were prepared using porous PGMA materials under identical formulation conditions. (C) Cross-sectional optical micrographs of porcine skin after microneedle insertion for each design. Yellow dashed lines delineate the insertion paths and deformation zones. Among all tested geometries, the 2 mm conical microneedles (design 3) exhibited optimal penetration depth and minimal tissue disruption, and were selected for subsequent studies. (D, E) SEM images of the porous structure of bare PMN (design 3).

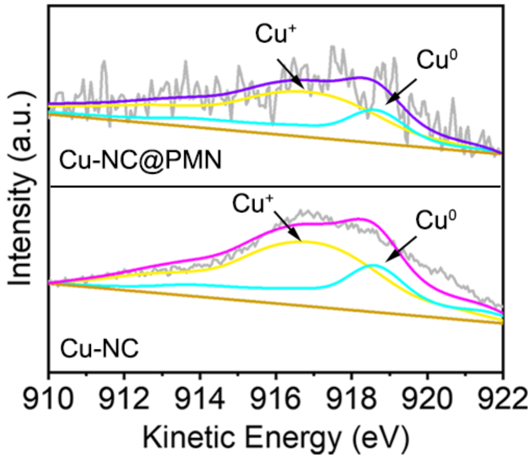

Fig. S10. Cu Auger LMM spectra of Cu-NC NBs and Cu-NC@PMN.

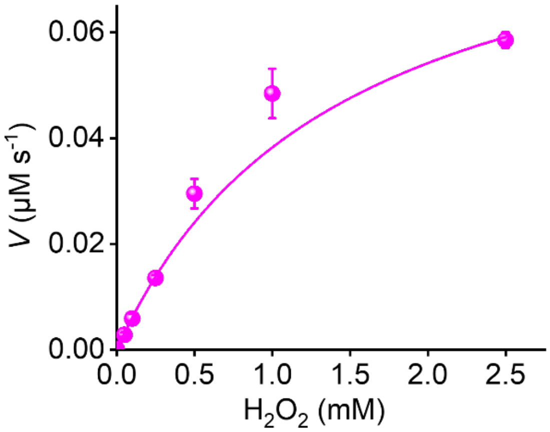

Fig. S11. Enzymatic kinetics for the POD-like activity of Cu-NC NBs (10 μg mL^{-1}).

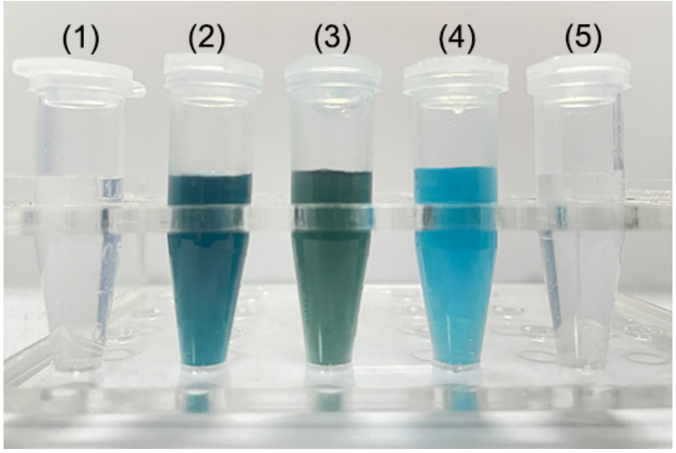

Fig. S12. Photograph of TMB oxidation in different groups: (1) H_2O_2, (2) H_2O_2 + HRP, (3) H_2O_2 + Cu-NC NBs, (4) H_2O_2 + Cu-NC@PMN, and (5) H_2O_2 + HRP@PMN.

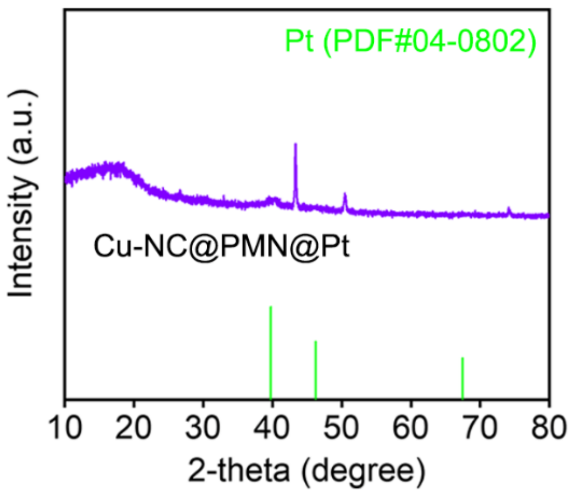

Fig. S13. **Powder XRD pattern of Cu-NC@PMN@Pt.**

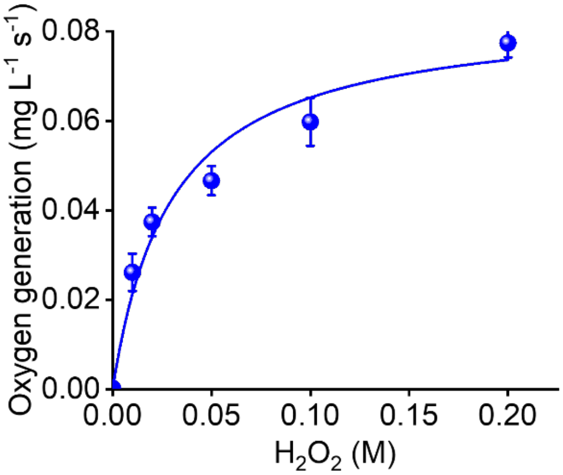

Fig. S14. Enzymatic kinetics of the CAT-like activity of Pt NPs (10 µg mL^{-1}).

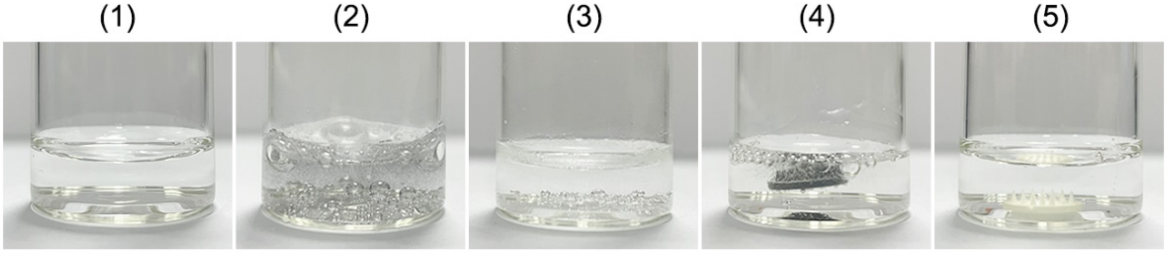

Fig. S15. Photograph of O_2 generation under different treatments: (1) H_2O_2, (2) H_2O_2 + Pt NPs (10 μg mL^{-1}), (3) H_2O_2 + CAT (10 μg mL^{-1}), (4) H_2O_2 + Pt@PMN, and (5) H_2O_2 + Cu-NC@PMN.

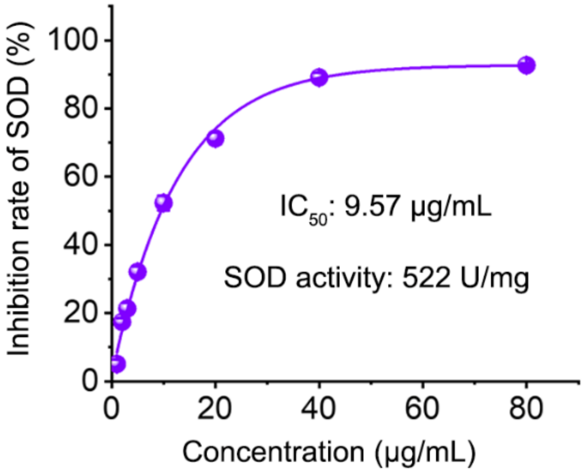

Fig. S16. Inhibition rate of WST-8 reduction by Pt NPs at different concentration.

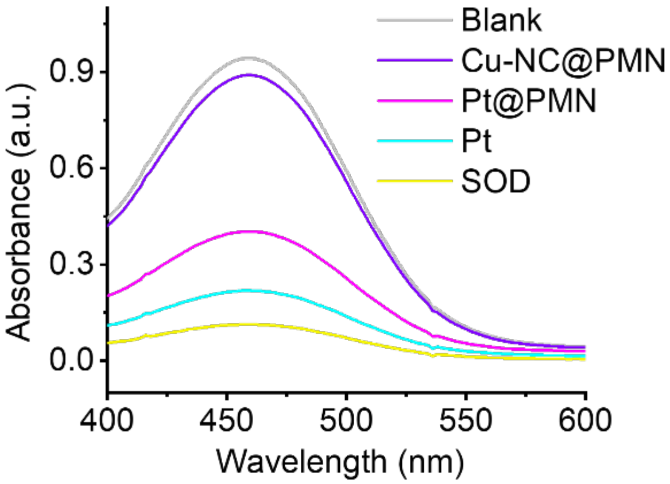

Fig. S17. UV-vis absorption spectra of WST-8 (a $\bullet O_2^-$ indicator) in $\bullet O_2^-$ solution containing various agents. $\bullet O_2^-$ was generated from the reaction of xanthine oxidase and xanthine.

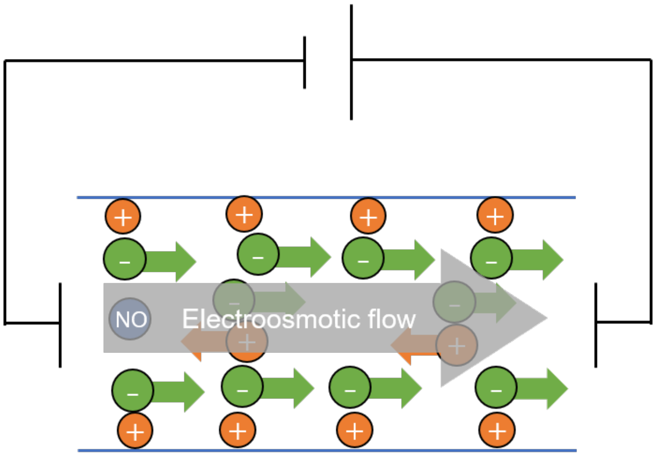

Fig. S18. Schematic illustration of the EOF mechanism within the positively charged porous microneedle channels. The inner surfaces of the microneedle pores are functionalized with positively charged groups, which electrostatically adsorb anionic counterions (e.g., Cl^{-} and HPO_4^{2-}) to form an anionic electric double layer (EDL). Upon application of an external electric field, these mobile anions are electrophoretically dragged toward the anode, generating a net fluid flow in the same direction. This electroosmotic flow enables directional transport of dissolved NO molecules from the interior catalytic region toward the skin interface for transdermal delivery.

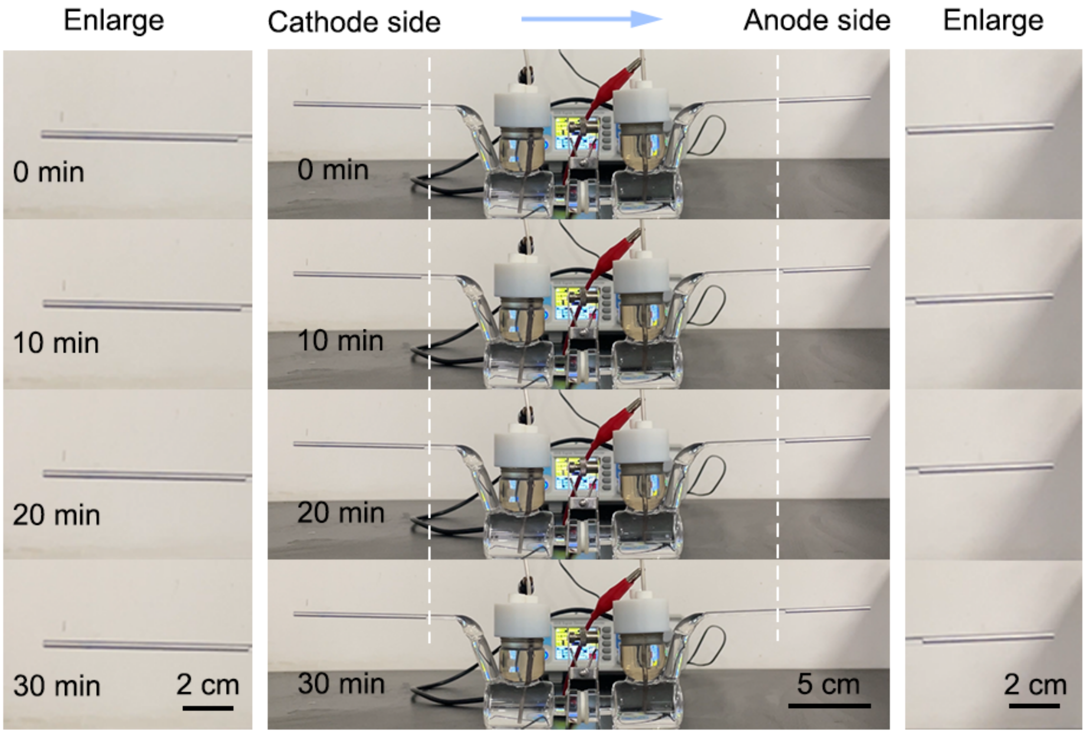

Fig. S19. Photographs of water migration within 30 min through the positively charged PMN under 1 V_{DC} power supply.

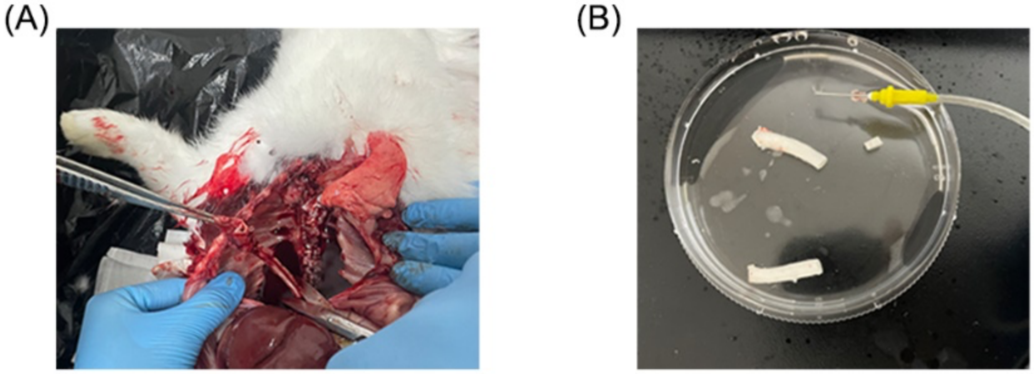

Fig. S20. Thoracic aorta extraction from rabbits. After euthanasia of the rabbit, the thoracic aorta was rapidly excised (A), placed in Krebs-Henseleit solution under physiological levels of O_2, and cut into a 5-mm-wide ring (B).

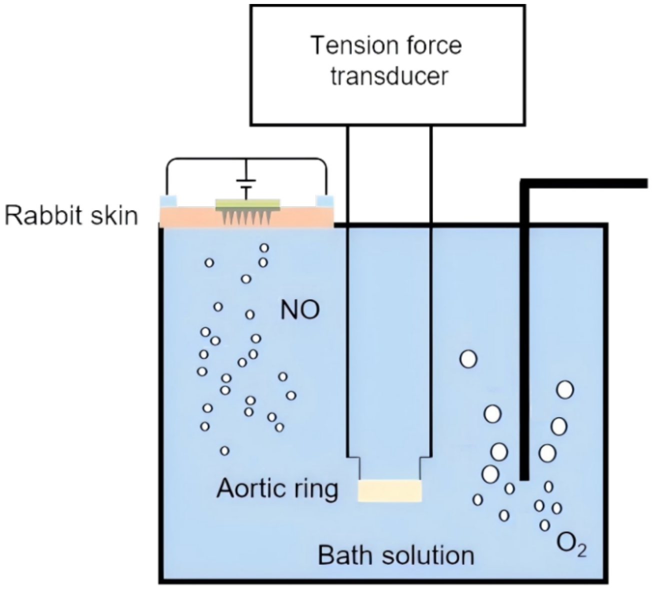

Fig. S21. Schematic of BPMS-induced vasodilation ex vivo. The aortic ring was attached to the force sensor and placed in 8 mL of Krebs-Henseleit solution at physiological O_2 level at 37 °C. The microneedle electrode of the BPMS was inserted into the rabbit skin to simulate transdermal delivery, with the other side of the skin in contact with the solution.

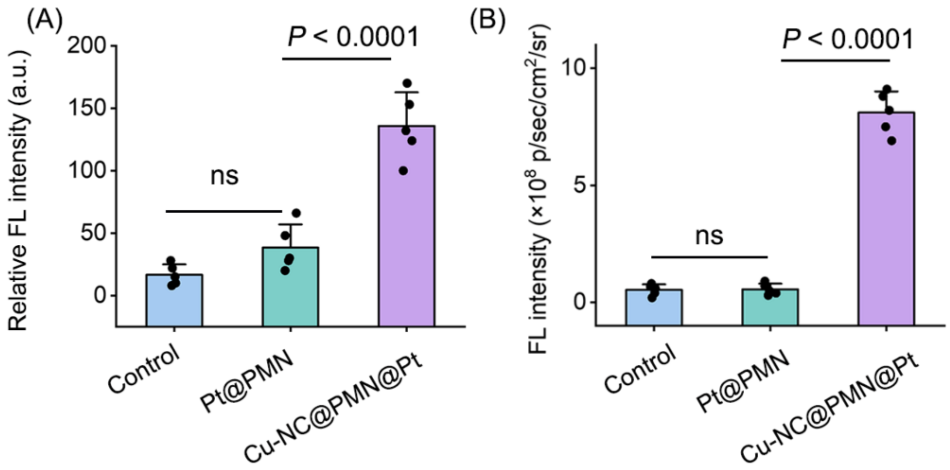

Fig. S22. Fluorescence intensity of vessel sections (A) and bulk vessels (B) with DAF-FM DA as a probe after the electrocatalytic NO generation of BPMS. Data are expressed as mean ± s.d, n = 5 biologically independent samples. One-way ANOVA and Tukey's post hoc test were used, and P values are labelled on the graphs. $^{*}P < 0.05$; $^{**}P < 0.01$; $^{***}P < 0.001$; $^{****}P < 0.0001$, ns denotes no significant difference ($P > 0.05$).

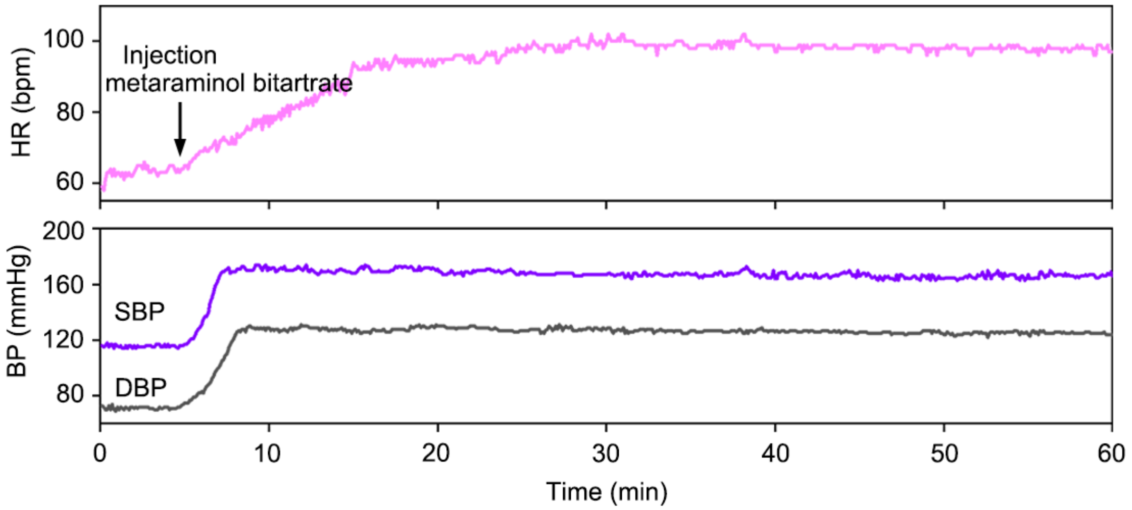

Fig. S23. Representative heart rate and blood pressure data of hypertensive pigs without BPMS treatment.

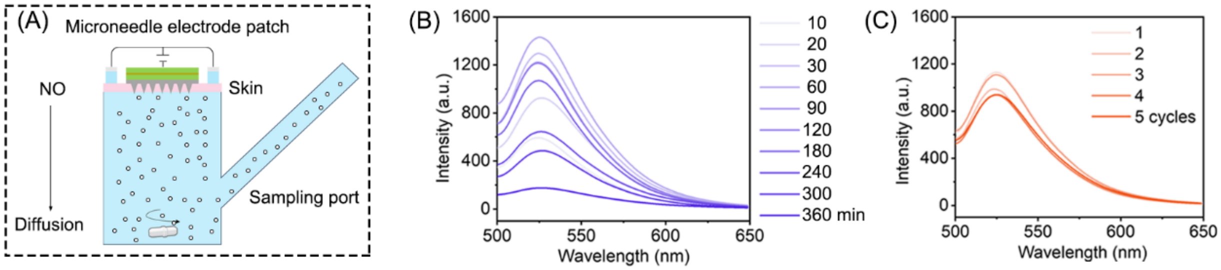

Fig. S24. Electrocatalytic NO generation performance and reusability of the microneedle electrode patch. (A) Schematic illustration of the in vitro setup for time-resolved NO release measurement using a Franz-type diffusion chamber. The Cu-NC@PMN@Pt microneedle electrode patch penetrates through a porcine skin layer to ensure direct contact between the electrode interface and the PBS buffer. NO is generated via electrocatalysis and diffuses into the buffer, where samples are collected from the side sampling port for analysis. (B) Time-resolved fluorescence spectra of NO released from the microneedle patch during continuous activation. DAF-FM was used as the NO probe (Ex/Em = 470/525 nm). Samples were taken at designated intervals over a 6-hour period, showing sustained NO production. (C) Reusability test of the microneedle electrode patch over five consecutive NO release cycles. After each cycle, the patch was rinsed and reloaded with fresh hydroxyurea solution (1 mM, 100 μL). Fluorescence intensity was recorded after 120 min of activation in each cycle. The NO signal shows minimal decrease across cycles, indicating excellent catalytic retention and mechanical durability of the Cu-NC@PMN@Pt microneedle array.

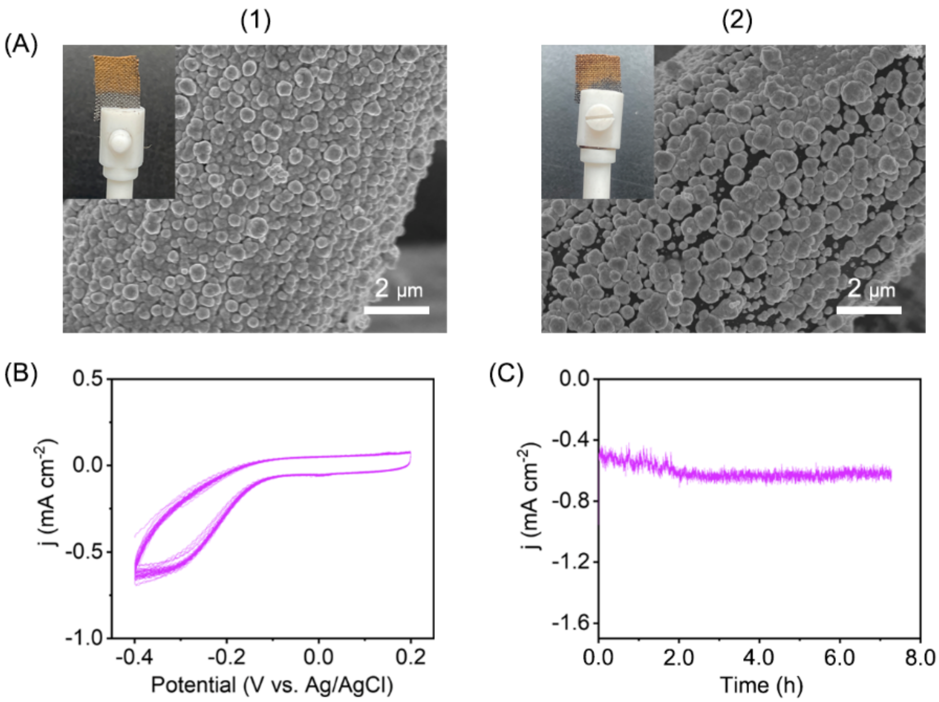

Fig. 25. Evaluation of Au NP adhesion and electrochemical durability. (A) SEM images of Au NP-deposited carbon cloth electrodes before (1) and after (2) complete device assembly. The optical photographs (top-left) show the electrode held in place using a custom electrode clip, which is used for imaging and subsequent electrode testing. No significant Au NP detachment or morphological change was observed following mechanical integration, indicating excellent structural robustness. (B) Multi-cycle cyclic voltammetry curves of the post-assembly Au NP electrode, demonstrating stable redox behavior. (C) Chronoamperometric (*I*–*t*) test of the post-assembly Au NP electrode under a constant potential of –0.3 V (vs. Ag/AgCl), showing long-term electrocatalytic stability. All electrochemical measurements were conducted in PBS buffer under O_2-saturated conditions (pH 7.4, 25 °C).

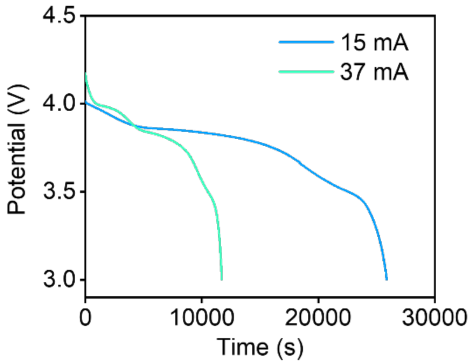

Fig. S26. Galvanostatic discharge profiles of a 100 mAh lithium-ion polymer battery under simulated system loads. Discharge was conducted at two constant currents: 15 mA (idle mode, blue) and 37 mA (working mode, green). The battery voltage was recorded over time, showing continuous operation for approximately 7.5 h and 3.3 h, respectively, before reaching the cutoff voltage of 3.0 V.

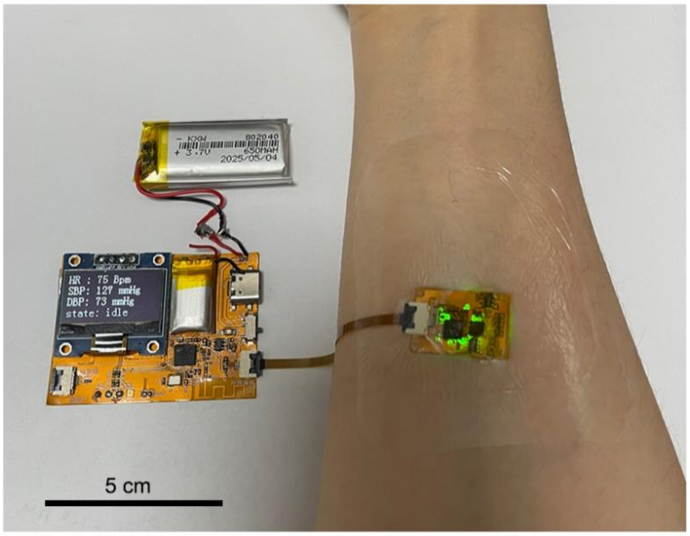

Fig. S27. Demonstration of the BPMS prototype upgraded with a high-capacity 650 mAh lithium-ion battery for extended operation. Compared to the original 100 mAh version, the upgraded 650 mAh battery significantly extends the system's runtime to ~45 h (idle mode) and ~20 h (work mode), supporting long-term wearable applications.

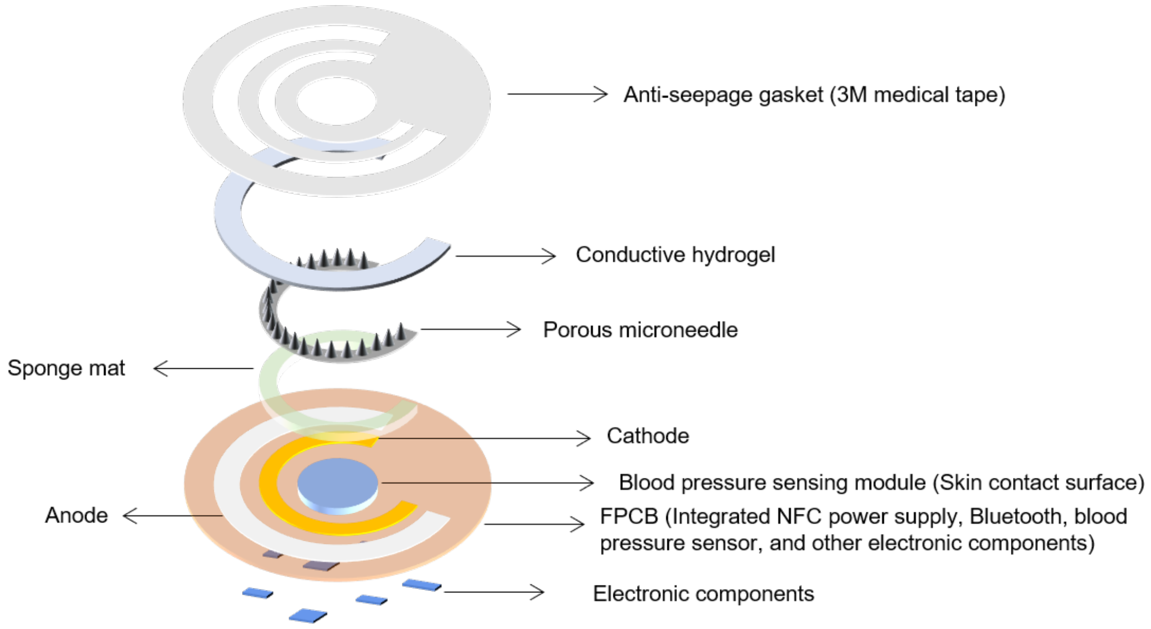

Fig. S28. Compact system design for future wearable BPMS. Exploded-view scheme of a miniaturized, skin-conformal BPMS integrating the microneedle actuator, sensing module, and control circuitry on a flexible platform. The OLED display is eliminated, and real-time output is displayed via a mobile app. Power is supplied wirelessly using NFC, supporting a battery-free, low-profile configuration suitable for daily wear.

Table S1. Average current consumption of key components in idle and work modes of BPMS.

Component	Idle mode current avg (mA)	Work mode current avg (mA)
ESP32 MCU (low duty)	~10	~20
BLE Transmission	~0.5	~0.5
BP sensing system	~4.5	~4.5
OLED Screen (Activated on user trigger or BP abnormal)	–	~10
Electrocatalysis LED indicator	–	~1
Electrode system	–	~1
Total (estimated)	~15	~37

To quantify system energy demand, we divided device operation into two primary modes:

- **Idle mode**: baseline blood pressure monitoring using PPG sensing and periodic Bluetooth communication.
- **Work mode**: full system activation, including BP monitoring, Bluetooth communication, OLED feedback, and electrocatalytic NO release.

The table summarizes the estimated current draw of major subsystems in both idle mode (BP sensing only) and work mode (BP sensing + electrocatalytic NO generation). These values are based on component datasheets and empirical measurements. The calculated average current supports a theoretical endurance of ~7 h (idle) and ~3 h using a 100 mAh lithium-ion polymer battery.
